# A Pair of Dopamine Neurons Target the D1-Like Dopamine Receptor DopR in the Central Complex to Promote Ethanol-Stimulated Locomotion in *Drosophila*

**DOI:** 10.1371/journal.pone.0009954

**Published:** 2010-04-01

**Authors:** Eric C. Kong, Katherine Woo, Haiyan Li, Tim Lebestky, Nasima Mayer, Melissa R. Sniffen, Ulrike Heberlein, Roland J. Bainton, Jay Hirsh, Fred W. Wolf

**Affiliations:** 1 Ernest Gallo Clinic and Research Center, Emeryville, California, United States of America; 2 Department of Anatomy, University of California San Francisco, San Francisco, California, United States of America; 3 Department of Biology, University of Virginia, Charlottesville, Virginia, United States of America; 4 Division of Biology, California Institute of Technology, Pasadena, California, United States of America; 5 Department of Anesthesia, University of California San Francisco, San Francisco, California, United States of America; UCLA, United States of America

## Abstract

Dopamine is a mediator of the stimulant properties of drugs of abuse, including ethanol, in mammals and in the fruit fly *Drosophila*. The neural substrates for the stimulant actions of ethanol in flies are not known. We show that a subset of dopamine neurons and their targets, through the action of the D1-like dopamine receptor DopR, promote locomotor activation in response to acute ethanol exposure. A bilateral pair of dopaminergic neurons in the fly brain mediates the enhanced locomotor activity induced by ethanol exposure, and promotes locomotion when directly activated. These neurons project to the central complex ellipsoid body, a structure implicated in regulating motor behaviors. Ellipsoid body neurons are required for ethanol-induced locomotor activity and they express DopR. Elimination of *DopR* blunts the locomotor activating effects of ethanol, and this behavior can be restored by selective expression of *DopR* in the ellipsoid body. These data tie the activity of defined dopamine neurons to D1-like DopR-expressing neurons to form a neural circuit that governs acute responding to ethanol.

## Introduction

Alcoholism is a major societal problem with great medical, financial and social costs. Environmental and genetic factors contribute to the susceptibility to alcoholism, and it is well established that the initial sensitivity of an individual to ethanol correlates with the likelihood of developing alcohol use disorders [1]. Similarly, breeding studies in rodents have identified positive correlations between the locomotor stimulant properties of acute ethanol exposure, a measure of ethanol sensitivity, and its reinforcing properties, suggesting that a genetic link for these traits may be evolutionarily conserved [2], [3]. Acute and repeated exposure to ethanol as well as to most other abused drugs increases dopamine (DA) levels in the mesolimbic region of the brain [4]; this brain region mediates many aspects of drug responding and reinforcement in a wide variety of behavioral paradigms [5]. For ethanol, mesolimbic DA is critically important for the regulation of drug sensitivity, consumption and preference [6]–[8]. Definition of the molecular, cellular, and neural circuit effects of acute ethanol exposure can provide a mechanistic understanding of how ethanol co-opts normal brain functions, and a foundation for understanding the complex shifts in behavior and physiology that underlie the development of addiction.

In the fruit fly *Drosophila melanogaster*, acute, repeated, and chronic exposure to ethanol, cocaine, or nicotine affects behavior in ways that appear similar to mammals [9]. Acute ethanol exposure stimulates locomotor activity at low doses, and promotes motor incoordination and sedation at higher doses [10], [11]. Repeated ethanol exposure results in sensitization to its locomotor activating effects and tolerance to its incoordinating and sedating effects [12]–[14], behavioral changes that can lead to increased ethanol intake in higher organisms. Moreover, flies show a preference for ethanol intake [15]. Importantly, DA signaling is critical for the actions of ethanol and cocaine in flies: disruption of DA signaling blunts the locomotor activating effects of ethanol, alters cocaine sensitivity, and decreases cocaine sensitization [16]–[18]. As in mammals, DA is also a pleiotropic modulator of behavior in flies, regulating arousal state, associative learning, and courtship behaviors [16], [19]–[24]. Understanding DA function at the molecular and circuit levels will help to define the relationships between these apparently distinct behaviors [25]–[28], and may provide insight into how drugs of abuse impinge upon natural behaviors.

**Figure 1:**
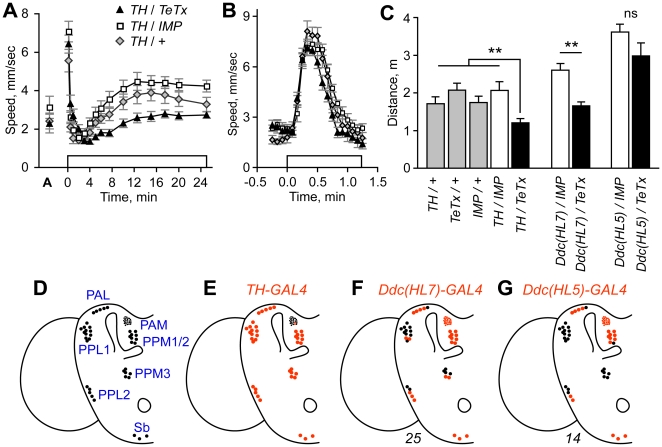
Blockade of evoked release from dopaminergic neurons reduced ethanol-induced hyperactivity. **A**. Expression of active tetanus toxin (TeTx) or an inactive form (IMP) as a control in tyrosine hydroxylase (TH) neurons (full genotype: *TH-GAL4*/+;*UAS-TeTx*/+). Open box indicates time of ethanol vapor exposure (47% ethanol vapor concentration). ‘A’ indicates locomotor speed of acclimated flies in humidified air just prior to ethanol exposure. **B**. Higher resolution analysis of the olfactory startle response. Peak speed achieved between 0–1 min ethanol exposure did not differ between genotypes (P = 0.2722, 1 way ANOVA, n = 10). **C**. Distance traveled for hyperactivity onset. *TH/TeTx* was different from indicated control genotypes (**P<0.01, 1-way ANOVA, Dunnett's multiple comparison test, n = 10). Expression of *TeTx* with *Ddc(HL7)* but not *Ddc(HL5)* resulted in reduced ethanol-induced hyperactivity (*Ddc(HL7)*: **P = 0.0006, *Ddc(HL5)*: P = 0.139, 2 sample t-test, n = 9–10). All transgenes were heterozygous in animals tested for behavior. **D**. Dopaminergic neuron cell body positions in one hemisphere of the adult brain. Drawing was adapted from Friggi-Grelin et al. [20]. DA neuron nomenclature describes the location of the cell bodies in the adult brain (for example the PPM3s are one of three clusters of protocerebral posterior medial DA cells) [30]. **E**–**G**. The average number of TH-positive cells that expressed the indicated GAL4 transgenes (red). Only PAM cell number is underrepresented. Number of hemispheres counted is indicated below the diagrams. The *TH-GAL4* expression pattern has been reported previously [20], [29].

The neuronal substrates for the effects of ethanol on behavior in *Drosophila* remain largely unknown. Defining the DA neurons that control ethanol-induced behaviors could provide a much needed entry point for future genetic and circuit analysis. In the fly brain approximately 140 DA neurons group into seven clusters in each hemisphere (**Fig. 1D**), and each DA neuron elaborates a complex projection that is stereotyped within a cluster [29], [30]. The connectivity and functional properties of the DA neurons are just beginning to be explored. The goal of this study was to determine if specific DA neurons and their targets in flies function in behavioral responses to acute ethanol exposure. Using genetic tools to inactivate or activate neurons, we identified a small set of DA neurons and their targets that promote locomotor stimulation by acute ethanol exposure. Additionally, we showed that the D1-like DA receptor DopR functions in the target neurons in the central complex, a brain region implicated in motor control. Our identification of specific DA and DA target neurons will allow detailed analysis, by genetic and other means, of circuit function in drug-induced and other behaviors, including those with rewarding properties. Moreover, circuit definition will help to place into context genes whose functions in drug abuse are less well understood.

## Results

### Dopaminergic neurons promote ethanol-induced locomotor activity

Flies placed in a behavioral testing chamber acclimate within 10 min and subsequently maintain low levels of activity (see *DopR* mutant characterization results below for details) [11]. Upon exposure to a continuous stream of ethanol vapor, acclimated flies immediately enter into a transient olfactory-mediated startle response (**Fig. 1A–B**). Following a brief period of quiescence, flies then exhibit a more sustained period of enhanced locomotor activity, the hyperactive phase, achieving peak speeds at 10–12 min. As flies continue to accumulate ethanol internally, they become progressively more uncoordinated, decrease overall locomotion, and eventually become sedated. The peak of the hyperactive phase corresponds to an inebriating 15–30 mM internal ethanol concentration [11]. Sedated flies recover following the termination of ethanol exposure [12]. The magnitude of the hyperactive phase is dose-dependent, with lower concentrations of ethanol vapor resulting in higher levels of hyperactivity, due in part to reduced sedation [11].

We asked if blockade of evoked neurotransmitter release from DA neurons in the CNS affected ethanol-induced hyperactivity. To do this, we expressed in DA neurons the tetanus toxin light chain (TeTx) that cleaves and inactivates the neuronal-specific isoform of synaptobrevin, blocking synaptic transmission [31]. This was accomplished by driving expression of the *UAS-TeTx* transgene with *TH-GAL4* that places the yeast transcriptional activator GAL4 under the control of the tyrosine hydroxylase (TH) locus [20], [31]. In *TH/TeTx* flies, ethanol-induced hyperactivity was reduced (**Fig. 1A**). Expression of catalytically inactive TeTx (IMP) had no effect, indicating that protein overexpression in TH neurons did not impinge upon neuron function. Determining the distance traveled during the onset of the hyperactive phase (2–12.5 min exposure) revealed a significant reduction in locomotor activity specifically in *TH/TeTx* flies (**Fig. 1C**). Similarly, locomotor speed achieved at 7.5 min of ethanol exposure was reduced in *TH/TeTx* flies (**Table S1**). However, using a higher resolution data analysis, we detected no change in the magnitude of the olfactory-mediated locomotor startle response of *TH/TeTx* flies (**Fig. 1B**). Additionally, locomotor coordination appeared normal in *TH/TeTx* flies, both by direct observation of fly movement, and in a negative geotaxis climbing assay, indicating that motor behaviors were grossly normal, and that at least one sensorimotor circuit was fully functional. Ethanol absorption was also unaffected (*TH/TeTx*: 38.3 mM, *TH/IMP*: 39.1 mM, 30 min at 47% ethanol vapor, P = 0.69, t-test, n = 3 groups of 25 flies each). Thus, blockade of evoked release from DA-producing neurons resulted in a specific decrease in the ethanol-induced stimulation of locomotor activity.

To confirm that disruption of DA neurotransmission was the basis for the observed ethanol-induced hyperactivity defect, we performed two additional experiments. First, the *Drosophila* DA transporter DAT is expressed in DA neurons, and flies mutant for DAT (*DAT^fmn^*) have been characterized for their altered sleep-like behavior [22], [32]. Flies homozygous for *DAT^fmn^* were viable, fertile, grossly normal for motor behaviors, and exhibited a normal olfactory startle response. Ethanol-induced hyperactivity, however, was markedly reduced in *DAT^fmn^* flies (**Fig. S1A**), indicating that disruption of DA reuptake and of evoked release from DA neurons had similar effects. Second, we confirmed that DA depletion by pharmacological means in adult flies reduced ethanol-induced hyperactivity (**Fig. S1B**) [16]. Thus, the effects of genetic and pharmacological manipulation of DA levels are consistent with the promotion of ethanol-induced hyperactivity by DA signaling in adult animals.!

### Role of PPM3 neurons in promoting ethanol-induced locomotor activity

We next asked whether DA signaling from many or a few neurons was required to promote ethanol-induced hyperactivity. As a first step, we built Dopa decarboxylase-GAL4 (*Ddc-GAL4*) transgenes that express GAL4 in subsets of TH- and 5-HT-positive neurons (**Fig. S1C**); Ddc executes the final step in DA synthesis and the locus is well characterized [33]. We then determined the behavioral effects of blocking evoked release in the patterns specified by the *Ddc* transgenes, and correlated the behavioral effects with expression in TH-positive neurons. Expression of TeTx with *Ddc(HL7)-GAL4* resulted in decreased ethanol-induced hyperactivity, whereas expression with *Ddc(HL5)-GAL4* did not (**Fig. 1C**, **Fig. S1E,G**). Previous studies demonstrated (and we confirmed) that *TH-GAL4* fully labeled all TH-positive clusters in the adult brain, except for the PAMs where approximately 12 of 100 cells showed expression (**Fig. 1D,E**) [20], [29]. With *Ddc(HL7)-GAL4*, fewer TH-positive cells were labeled in the PAL, PPL1, PPL2, and PPM3 clusters (**Fig. 1F** **, Fig. S1D**). *Ddc(HL5)-GAL4* expression was further restricted, with no expression detected in the PPL1 and PPM3 clusters (**Fig. 1G** **, Fig. S1F**). We also detected 1–2 fewer labeled cells in the PPL2 and PPM1/2 clusters in *Ddc(HL5)-GAL4*. In the thoracic ganglion, nearly all TH-positive neurons were labeled by *Ddc(HL5)-GAL4* (**Fig. S2A**), indicating that thoracic DA neurons were not necessary to promote ethanol-induced hyperactivity. These data suggested that DA promotion of ethanol-induced hyperactivity was localized to specific subsets of DA neurons, or, alternatively, that activity in a substantial fraction of DA neurons was required for normal ethanol responses.

**Figure 2:**
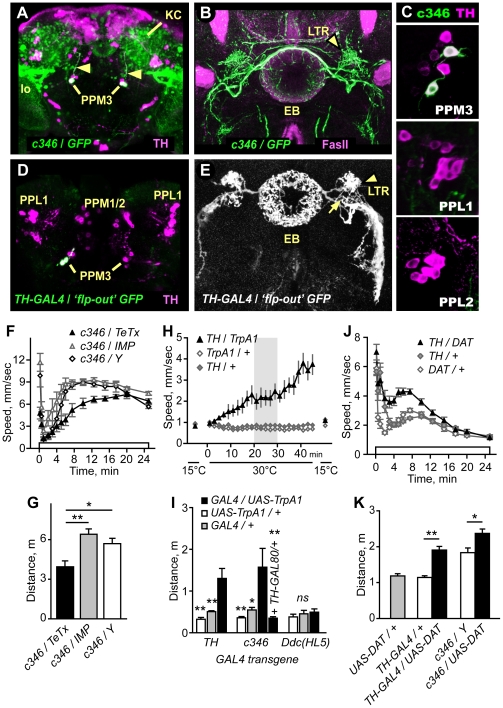
TH-positive PPM3 neurons that project to the ellipsoid body promote ethanol-induced hyperactivity. **A**. *c346* (detected by *UAS-GFP*, green) was expressed in two TH-positive (magenta) PPM3 neurons per hemisphere. The *c346* PPM3 processes projected anteriorly (arrowheads) towards the central complex. *c346* was also detected in the mushroom body Kenyon cells (KC) and in neurons that projected to the lobula (lo). **B**. *c346* (green) processes project to the lateral triangles (LTR) and ring of the ellipsoid body (EB), counterstained with FasII (magenta). **C**. *c346* expression in TH-positive cells is limited to the PPM3s. **D**,**E**. Labeling of individual *TH-GAL4* cells using the flp-out technique. Two GFP-labeled TH cells in the PPM3 cluster projected anteriorly to the LTR region (entry point is marked with an arrow in E). The process branched to innervate the EB ring and LTR, and the ventral body region. **F**,**G**. Distance traveled was reduced in *c346/TeTx* (*P<0.05, **P<0.01, 1-way ANOVA, Tukey's multiple comparison test) whereas the startle response was not (P = 0.0925, 1-way ANOVA, n≥6). **H**. Transient activation of TH neurons increased locomotor activity. Expression of the heat-activated TrpA1 ion channel in TH neurons at 15°C (off) and 30°C (on). **I**. Distance traveled from 20–30 min at 30°C for *TH/TrpA1* (**P<0.01, 1-way ANOVA, Dunnett's multiple comparison test, n≥5). *c346/TrpA1* also increased locomotor activity. Flies of the genotype *c346/Y;TH-GAL80/+;UAS-TrpA1/+*, where GAL4 activity was blocked by GAL80 solely in *c346* PPM3 neurons, showed no increase in locomotor activity (*P<0.05, **P<0.01, 1-way ANOVA, Dunnett's multiple comparison test to *c346*/*Y*; *UAS-TrpA1*/+, n≥6). Activation of TrpA1 in the *Ddc(HL5)* pattern had no effect (P = 0.6243, 1-way ANOVA, n≥5). **J**,**K**. Overexpression of DAT in *TH-GAL4* or *c346* neurons increased ethanol-induced hyperactivity. (*P = 0.0123, **P = 0.0005, 2 sample t-test, n≥10).

To test the possibility that discrete DA neurons regulated ethanol-induced hyperactivity, we searched for GAL4 transgenic lines that expressed specifically in the DA neurons present in *Ddc(HL7)-GAL4* and absent in *Ddc(HL5)-GAL4*. One GAL4 line, *c346*, was expressed in two of the 6–8 TH-positive PPM3 neurons and in no other TH-positive neurons (**Fig. 2A–C**). *c346* sparsely labeled other neurons in the adult CNS, including a subset of the mushroom body Kenyon cells, a group of cells located in the lateral protocerebrum that projected to the lobula of the optic lobe, and approximately 15–20 cells in the thoracic ganglion (**Fig. S2B**). The two TH-positive *c346*-expressing PPM3 cell bodies, located at the posterior surface of the brain, extended a fasciculated process anteriorly towards the central complex (**Fig. 2B** **, Movie S1**). The central complex includes four interconnected neuropils: the ellipsoid body, fan-shaped body, noduli, and the protocerebral bridge. Specific regions of the central complex are heavily innervated by dopaminergic processes [29], [30], [34]. The *c346*-labeled PPM3 processes branched at the central complex, and elaborated dense networks of processes in three regions: the central complex ellipsoid body ring and lateral triangles, and the ventral body region located ventral and lateral to the central complex.

To confirm that PPM3 neurons innervated the ellipsoid body, we used the ‘flp-out’ technique to randomly label subsets of neurons in the *TH-GAL4* expression pattern. All PPM3 neurons detected by this method innervated the central complex. Three distinct patterns of innervation were found: dense arborization in a discrete layer of the fan-shaped body either with or without additional arborization in the noduli (not shown), and dense arborization in the ellipsoid body ring and lateral triangles (**Fig. 2D,E**). This latter innervation pattern appeared identical to that observed in *c346*. These innervation patterns, also observed in a recent survey of DA neuron projection patterns [29], indicate that the PPM3 DA neurons make extensive connections in the central complex, and that *c346* specifically labels PPM3 neurons that project to the ellipsoid body. We detected no innervation of the central complex by other TH-positive neurons. However, one PPL1 neuron has been reported to make an arborization in the fan-shaped body [29]. *Ddc(HL7)* but not *Ddc(HL5)* also showed expression in neurons that innervated the ellipsoid body, suggesting that *Ddc(HL7)* and *c346* labeled the same subset of PPM3 neurons (**Fig. S3**).

Importantly, expression of TeTx but not IMP in the *c346* pattern resulted in reduced ethanol-induced hyperactivity (**Fig. 2F,G**), indicating that evoked release from neurons in the *c346* expression pattern regulated ethanol-induced hyperactivity, and suggesting that the dopaminergic PPM3 neurons may mediate this effect.

### PPM3 neurons regulate locomotor activity

If DA release in a subset of DA neurons promotes ethanol-induced hyperactivity, then directly activating these neurons may also increase locomotor activity. To address this possibility, we expressed the heat-activated TrpA1 ion channel (*UAS-TrpA1*) in *TH-GAL4* or *c346* neurons, and assessed the behavioral effects of transient neuronal activation [35]. At 15°C, when TrpA1 is inactive, *TH/TrpA1* flies showed low levels of locomotor activity (**Fig. 2H**). When the temperature was raised to 30°C, above the temperature required for TrpA1 activation, *TH/TrpA1* flies increased locomotor activity levels, whereas control flies did not (**Fig. 2H,I**). Interestingly, returning *TH/TrpA1* flies to 15°C almost immediately resulted in a cessation of locomotion. Moreover, repeated temperature cycling caused repeated cycling between states of inactivity and activity (not shown), suggesting that locomotor activity may be specified by continued signaling by DA neurons. These data indicated that acute activation of DA neurons promotes locomotor activity.

When TrpA1 was activated in *c346*-expressing neurons, flies also increased locomotor activity (**Fig. 2I**). Importantly, activation of neurons in the *Ddc(HL5)* pattern had no effect. Thus, the stimulation of locomotor activity by activation of either *TH-GAL4* or *c346* neurons was specific, and the subset of DA neurons labeled by the *Ddc(HL5)* transgene (PAL, PAM, PPL2, PPM1/2, Sb) could not be the sole source of a DA-dependent locomotor activation signal. To determine whether the stimulation of locomotor activity by *c346* neurons was due to activation of the PPM3s, we included *TH-GAL80* to block *c346* GAL4 activity specifically in TH-expressing neurons. In *c346;TH-GAL80/+;UAS-TrpA1/+* flies, no stimulation of locomotor activity was detected (**Fig. 2I**). These data indicate that the dopaminergic PPM3 neurons that project to the ellipsoid body ring and lateral triangle promote locomotor activity when stimulated.

Lastly, we asked whether increasing DAT levels in DA neurons affected locomotor activity. Overexpression of DAT in either the *TH-GAL4* or the *c346* pattern resulted in increased ethanol-induced hyperactivity (**Fig. 2J,K**), the opposite behavioral response as compared to the loss-of-function *DAT^fmn^* mutants (**Fig. S1A**). Taken together, these data suggest that ethanol-induced stimulation of locomotor activity was due at least in part to DA signaling from PPM3 neurons that project to the ellipsoid body ring and lateral triangle region of the brain.

### Central complex ellipsoid body neurons promote ethanol-induced locomotor activity

The central complex, the putative downstream target of the PPM3 DA neurons, plays multifaceted roles in the regulation of movement behaviors, including locomotion and flight (for review, see [36] and it has been implicated in the development of ethanol tolerance [14], [37]. To determine if the central complex contributes to ethanol-induced hyperactivity, we expressed TeTx in neuronal patterns dictated by a collection of 24 GAL4 driver lines that showed expression in discrete subsets of central complex neurons (*CC-GAL4*) (**Table 1**). The phenotypes of *CC-GAL4/TeTx* flies fell into four classes: non-viable, non-responsive (no startle or hyperactive phase), defective in ethanol-induced hyperactivity, and unaffected. There was no obvious correlation between TeTx-induced lethality and central complex expression pattern, suggesting that evoked release from the central complex is non-essential. Six of the seven non-responsive class *CC-GAL4* lines expressed GAL4 either in the fan-shaped body or in small field neurons that connect different regions of the central complex. This finding is consistent with previous evidence for a role of the central complex in locomotion [38].

**Table 1:** Expression of TeTx in GAL4 patterns that include the central complex.

GAL4 Line	GAL4/TeTx Class	CC Expression
*4.67*	Hyperactivity defective	EB R2/R4
*5.30*	Hyperactivity defective	EB R2/R4
*11.148*	Hyperactivity defective	EB R2/R4
*c819/c547*	Hyperactivity defective	EB R2/R4m
*3.16*	Unaffected	FSB
*4.13*	Unaffected	FSB
*4.69*	Unaffected	EB R neurons
*11.17*	Unaffected	EB R1/R3
*189Y*	Unaffected	EB R3
*c232*	Unaffected	EB R3/R4d
*c561*	Unaffected	EB R1
*11.33*	Non-responsive	FSB
*11.243*	Non-responsive	FSB
*12.30*	Non-responsive	FSB
*78Y*	Non-responsive	Small-field
*c42*	Non-responsive	EB R2/R4m, FSB
*c107*	Non-responsive	Small field
*c161*	Non-responsive	Small field
*2.70*	Non-viable	FSB
*5.21*	Non-viable	EB R4
*5.36*	Non-viable	EB R1 or R3
*6.61*	Non-viable	EB R2/R4
*11.32*	Non-viable	EB R neurons
*11.113*	Non-viable	EB R Neurons

Abbreviations: CC, central complex; EB, ellipsoid body; FSB, fan shaped body.

**Figure 3:**
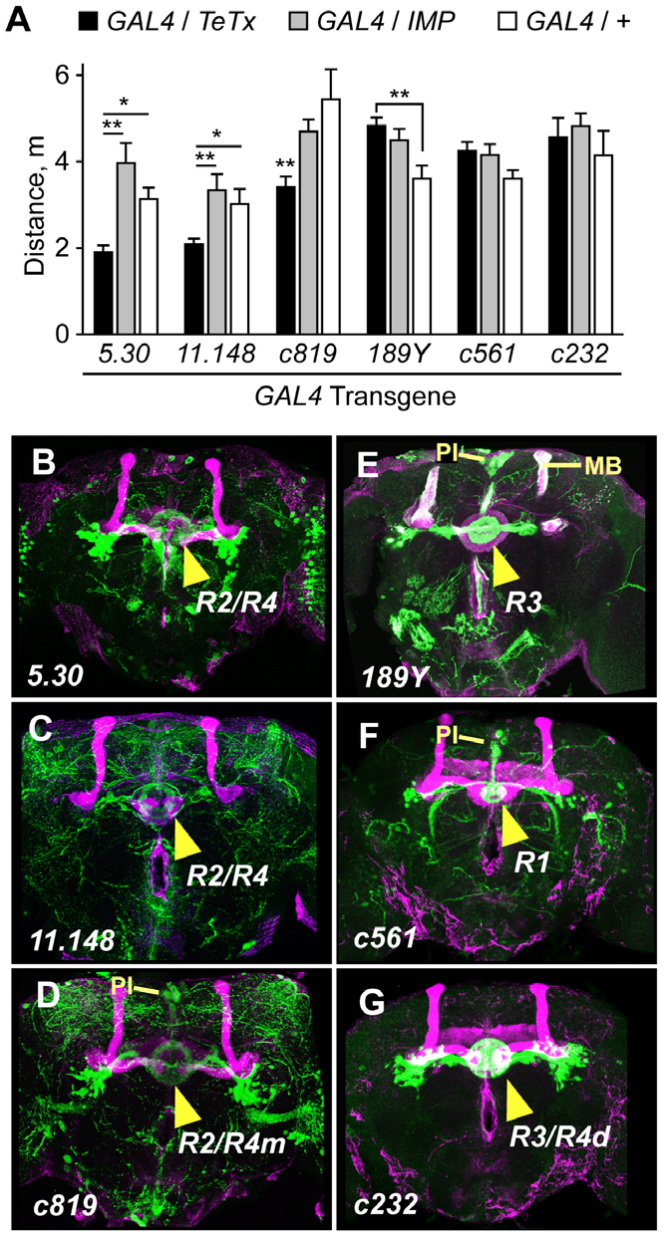
Functional mapping of ellipsoid body neurons in ethanol-induced hyperactivity. Blockade of evoked release in patterns that included R2/R4 neurons of the ellipsoid body (**B**–**D**, GAL4 transgenic lines *5.30*, *11.148*, and *c819*) resulted in reduced ethanol-induced hyperactivity (**A**). Ethanol-induced hyperactivity was not reduced by TeTx expression in patterns that included R1 (*c561*), R3 (*189Y*, *c232*), or R4d (*c232*) neurons of the ellipsoid body (**E**–**G**). *P<0.05, **P<0.01, 1-way ANOVA for a given GAL4 transgene, Tukey's multiple comparison test, n≥5. For *c819*/*TeTx* vs. *c819/IMP*, **P = 0.0041, 2 sample t-test, n = 8. **B**–**G**. Expression of *UAS-GFP* (green) by the indicated GAL4 transgene, counterstained with FasII antibodies (magenta). Arrowheads indicate innervation of the ellipsoid body ring. PI: pars intercerebralis, MB: mushroom bodies.

Four *CC-GAL4* lines (*4.67*, *5.30*, *11.148*, and *c819/c547*) resulted in reduced ethanol-induced hyperactivity when crossed to TeTx (**Fig. 3A**, **Fig. S4A–C**). All four lines showed expression in the R2/R4 subset of ellipsoid body neurons (**Fig. 3B–D** **, Fig. S4G,H**). These lines also showed expression in other regions of the brain and in the thoracic ganglion (**Fig. S2**). However, we detected no clearly discernible overlap of expression outside of the ellipsoid body (see below). One line, *4.67*, was also expressed in a subset of TH-positive neurons. Finally, eight *CC-GAL4* lines showed no apparent behavioral defect when crossed to *UAS-TeTx* (**Fig. 3A**). Three of these eight lines (*189Y*, *c232*, *c561*) were expressed in ellipsoid body neurons belonging to the R1, R3, and R4d classes (**Fig. 3E–G** **, Figure S4D–F**). Taken together, these data demonstrate a strong correlation between blocking evoked release in ellipsoid body neurons of the R2/R4 class and reduced ethanol-induced hyperactivity.

Other brain regions implicated in locomotor control or ethanol responses appear to be dispensable for the promotion of ethanol-induced hyperactivity. First, the mushroom bodies regulate overall locomotor activity levels, but not sensitivity to the incoordinating and sedating effects of ethanol [39], [40]. Similarly, we found that ethanol-induced hyperactivity was unaffected following chemical ablation of the mushroom bodies (**Fig. S5**). Furthermore, the GAL4 line *189Y* was expressed in the mushroom bodies and showed no phenotype when crossed to TeTx (**Fig. 3E**) [41]. Finally, we were unable to rescue the ethanol hyperactivity phenotype of *DopR* mutants utilizing mushroom body GAL4 drivers (see below). These data suggest that the mushroom bodies are not involved or play a redundant role in ethanol-induced hyperactivity. Second, the thoracic ganglion contains sufficient circuitry for some motor programs, including coordinated locomotion [42], [43]. We exposed freshly decapitated flies to ethanol vapor to ask if ethanol directly stimulates thoracic ganglion locomotor circuits. Decapitated flies maintained the righting response and the ability to groom, and walked a few steps when prodded, indicating that thoracic ganglion circuitry was functional. However, ethanol exposure elicited no increase in locomotor activity (**Fig. S2E**). Finally, neurosecretory cells of the pars intercerebralis regulate ethanol sensitivity [44]. However, pars intercerebralis expression in the *CC-GAL4* lines did not correlate with TeTx behavioral phenotypes in our experiments (**Fig. 3D–F**). Collectively, our findings are consistent with a model whereby evoked neuronal activity in specific regions of the central complex, most likely the R2 and/or R4 neurons of the ellipsoid body, promotes ethanol-induced hyperactivity.

### D1-like DopR receptor is expressed in the ellipsoid body

**Figure 4:**
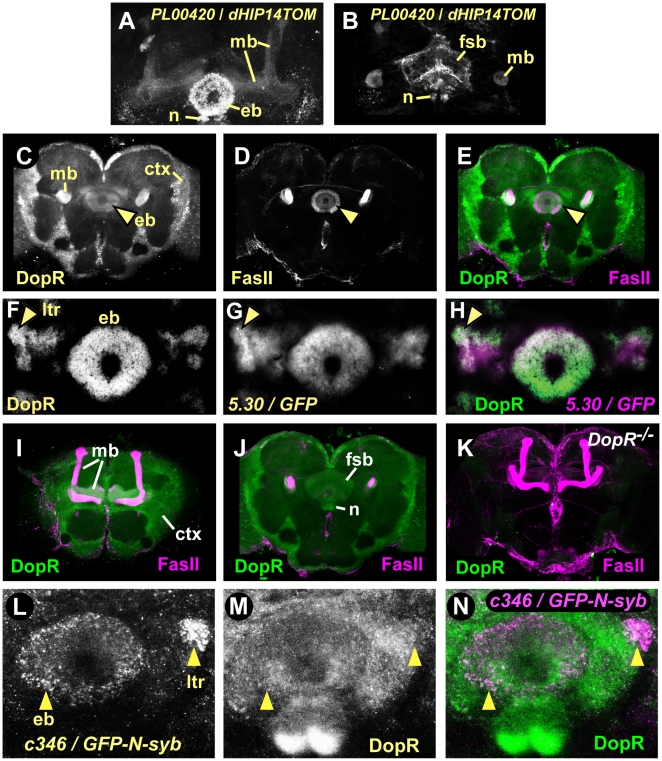
The D1-like DA receptor DopR expression pattern in the adult brain includes the R neurons of the central complex ellipsoid body. **A**–**B**. Presynaptic regions of *DopR*-expressing neurons. GAL4 enhancer trap *PL00420* in the *DopR* locus driving expression of tdTomato-GFP tagged with presynaptic protein HIP14. **A**. Expression in the mushroom body lobes (mb), ellipsoid body neurons (eb), and central complex noduli (n). **B**. Expression in the central complex fan shaped body (fsb) and noduli, and the mushroom body peduncles. **C**–**E**. Genetic background control brains stained with DopR (green) and FasII (magenta) antisera. Each panel is a 4.3 µm confocal section of a whole mount brain. Ellipsoid body (arrowhead), mushroom body peduncles, and cellular cortex (ctx) labeling with DopR antisera. **F**–**H**. Ellipsoid body lateral triangles (arrowhead) and ring showed elevated DopR levels that were coincident with the *5.30* GAL4 enhancer trap expression pattern in 10 µm thin sections. **I**. Mushroom bodies and cellular cortex staining in controls. **J**. Fan-shaped body and noduli DopR staining in controls. **K**. *DopR^f02676^* mutant brain (*DopR^−/−^*) stained with DopR (green) and FasII (magenta) antisera. 100 µm confocal projection shows nearly absent DopR staining throughout the brain. **L–N**. Colabeling of *c346* presynaptic sites (using n-syb-tagged GFP) and DopR in the ring and lateral triangles.

The D1-like DA receptor DopR (DmDop1, dDA1) is present in the central complex and the mushroom bodies [27], [45]. To ask if DopR is present in the ellipsoid body, we first determined the expression pattern of a GAL4 enhancer trap, *PL00420*, that resides in the first intron of the *DopR* locus (see below). Brains labeled with *PL00420/UAS-dHIP14tdTOM* (preferentially labeling presynaptic sites of neurons [46]) showed robust expression in the central complex ellipsoid body ring, noduli, and fan shaped body (**Fig. 4A,B**). We also observed labeling of the mushroom bodies, subsets of antennal lobe glomeruli and other less well-defined regions of the protocerebrum. Furthermore, we stained brains of our genetic background control strain with antiserum raised against a peptide derived from the third intracellular loop of DopR (**Fig. 4C–J**). DopR protein was expressed in the ellipsoid body ring (**Fig. 4C–E**, arrowhead), fan shaped body, noduli (**Fig. 4J**), and mushroom bodies (**Fig. 4I**). These patterns were specific, as all immunostaining was dramatically reduced in *DopR* mutant flies that expressed very low levels of *DopR* transcript (see below) (**Fig. 4K**). Furthermore, DopR was present in both the ring and lateral triangles of the ellipsoid body, where DopR staining overlapped with neuronal processes of R2/R4 neurons labeled by *5.30-GAL4* (**Fig. 4F–H**). Finally, presynaptic terminals of the *c346* PPM3 neurons required for ethanol-induced hyperactivity overlapped with or were closely apposed to DopR in the ellipsoid body and the lateral triangles (**Fig. 4L–N**). Thus, the D1-like DopR receptor is expressed in many structures throughout the adult brain, including the R2/R4 neurons of the ellipsoid body, and these neurons may be in synaptic contact with the PPM3 neurons.

### DopR is required for ethanol-induced hyperactivity

**Figure 5:**
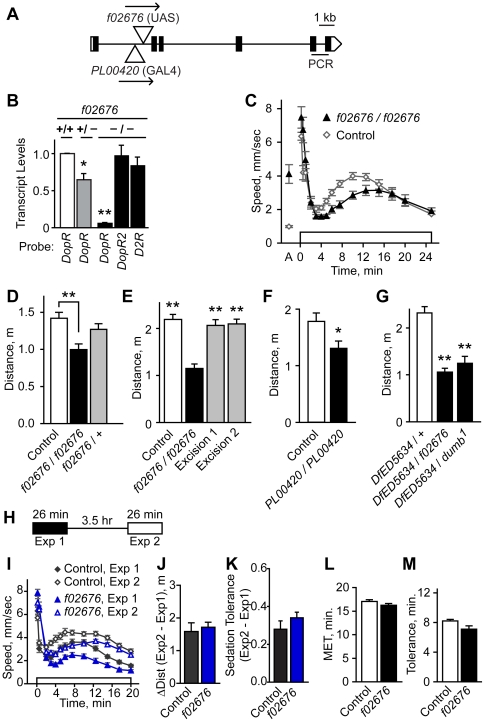
Molecular and behavioral characterization of *DopR* mutants. **A**. *DopR* gene structure. Positions of the transposons *f02676* and *PL00420* are indicated in the *DopR* locus. Arrows indicate orientation of *UAS* sites for *f02676* and *GAL4* for *PL00420*. Black rectangles indicate the single predicted open reading frame. **B**. Relative to genetic background controls, *DopR* transcript levels were nearly eliminated in *DopR^f02676^* homozygotes (−/−), whereas transcript levels of the DA receptors *DopR2* and *D2R* were unchanged, as determined by quantitative RT-PCR (*P<0.05, **P<0.01, 1 way ANOVA, Dunnet's multiple comparison test to control, n = 3 replicates). *DopR* PCR probeset location is indicated in A. **C**, **D**. Ethanol-induced locomotor activity was reduced in *DopR^f02676^* homozygotes (**P<0.01, 1 way ANOVA, Tukey's multiple comparison test, n≥11). **E**. Precise excision of *f02676* reverted the ethanol-induced hyperactivity phenotype (**P<0.01, 1 way ANOVA, Dunnett's multiple comparison test, n = 12). **F**. Reduced ethanol-induced hyperactivity in *DopR^PL00420^* homozygotes (*P = 0.0116, 2 sample t-test, n = 11). **G**. Non-complementation for ethanol-induced hyperactivity by *DfED5634*, a deficiency that deletes the entire *DopR* locus, for the *DopR f02676* and the *dumb^1^* inversion alleles (**P<0.01, 1 way ANOVA, Dunnett's multiple comparison test, n≥6). **H–K**. *DopR* mutants develop normal ethanol tolerance. **H**. Ethanol exposure scheme to induce and measure rapid ethanol tolerance. Flies were exposed twice for 26 min each to ethanol vapor (60%), separated by a 3.5 hr rest period. Locomotor activity was quantified for both exposures, and the number of flies sedated was counted immediately after each exposure. **I**. Locomotor activity profiles for exposure 1 and 2 for genetic background control and *DopR^f02676^* flies. **J**. No change in distance traveled was detected from 2–25 min, Exposure 2 minus Exposure 1 (ΔDist) (P = 0.6691, paired t-test, n = 15). **K**. Sedation tolerance, the fraction awake exposure 2 minus exposure 1, was unaffected (P = 0.27, 2 sample t-test, n = 15). **L**. Ethanol sensitivity as measured in the inebriometer was unaffected (P = 0.16, 2 sample t-test, n = 5). **M**. Rapid tolerance, as measured in the inebriometer, was unaffected (P = 0.09, 2 sample t-test, n = 5).

To ask if DopR contributes to ethanol-induced hyperactivity, we tested the effects reducing the levels of *DopR*. *DopR^f02676^* is a transposon insertion allele tha thet dramatically reduces DopR protein levels [21] (**Fig. 4K** **,** **5A**). In agreement with this, we found that *DopR* transcript was reduced by 95% in the heads of flies homozygous for *DopR^f02676^* (**Fig. 5B**). No compensatory changes were detected in transcript levels for the DA receptors *DopR2* and *D2R*. Similar to the effects of blocking evoked neurotransmission from dopaminergic and ellipsoid body neurons, *DopR^f02676^* mutants showed reduced ethanol-induced hyperactivity (**Fig. 5C,D**), and no change in ethanol sedation sensitivity (**Fig. S6A**) or ethanol absorption (*DopR^f02676^*: 35.2 mM, control: 36.3 mM, 30 min at 47% ethanol vapor, P = 0.66, t-test, n = 6). Precise excision of *f02676* restored both *DopR* transcript levels and ethanol-induced hyperactivity levels to those of controls, indicating that the *f02676* transposon insertion was responsible for the molecular and behavioral phenotypes (**Fig. 5E** **, Fig. S6C**). Flies homozygous for the *PL00420* transposon insertion showed 30% reduced *DopR* transcript levels and reduced ethanol-induced hyperactivity (**Fig. 5F** **, Fig. S6E**). Moreover, either *f02676* or the *dumb^1^* inversion breakpoint allele of *DopR* placed in *trans* to a deficiency that deleted the *DopR* locus showed reduced ethanol-induced hyperactivity (**Fig. 5G** **, Fig. S6G**). Taken together, these data demonstrate that decreased levels of the DopR receptor resulted in reduced ethanol-induced hyperactivity. Because the effects of the *DopR* mutations on ethanol-induced locomotor activation were partial, other DA receptors may also play a role in this behavior.

*DopR^f02676^* mutants also showed increased activity prior to ethanol exposure (‘A’ in **Fig. 5C**). While opposite in sign, increased pre-exposure activity could influence the magnitude of the decrease in ethanol-induced hyperactivity in *DopR^f02676^*. To quantify this behavior, we recorded the locomotion of flies without an added stimulus (‘unstimulated activity’) (**Fig. S6B**). Immediately after being placed in the observation chamber, control flies showed moderate levels of activity (4.7 mm/sec), and they quickly acclimated to their environment, maintaining low levels of activity for at least 1 hr (1.2–2.5 mm/sec). *DopR^f02676^* mutants showed higher initial levels of activity (8.6 mm/sec), and while *DopR^f02676^* flies also acclimated, they maintained two-fold higher activity levels (4.9–5.3 mm/sec) (**Fig. S6B**). This phenotype was due to the *f02676* insertion (**Fig. S6D**). However, unstimulated activity was unaffected in *DopR^PL00420^* homozygotes (**Fig. S6F**). Moreover, we previously demonstrated a lack of correlation between pre-exposure locomotor activity and ethanol-induced hyperactivity levels [11]. Thus, flies with strongly reduced *DopR* levels showed increased unstimulated activity and this behavior could be dissociated from the role of *DopR* in ethanol-induced hyperactivity.

Ethanol tolerance is a form of acquired resistance that facilitates increased drug intake, a major risk factor for the development of alcohol use disorders [47]. The development of ethanol tolerance in flies involves central complex neurons and the ellipsoid body [14], [37]. We found that *DopR^f02676^* mutants performed indistinguishably from genetic background controls in a rapid tolerance paradigm, showing both increased ethanol-induced hyperactivity and increased resistance to the sedating effects of ethanol during a second exposure to ethanol vapor (**Fig. 5H–M**). Thus, *DopR* is not required for rapid ethanol tolerance.

### DopR functions in the ellipsoid body for ethanol-induced locomotor activity

**Figure 6:**
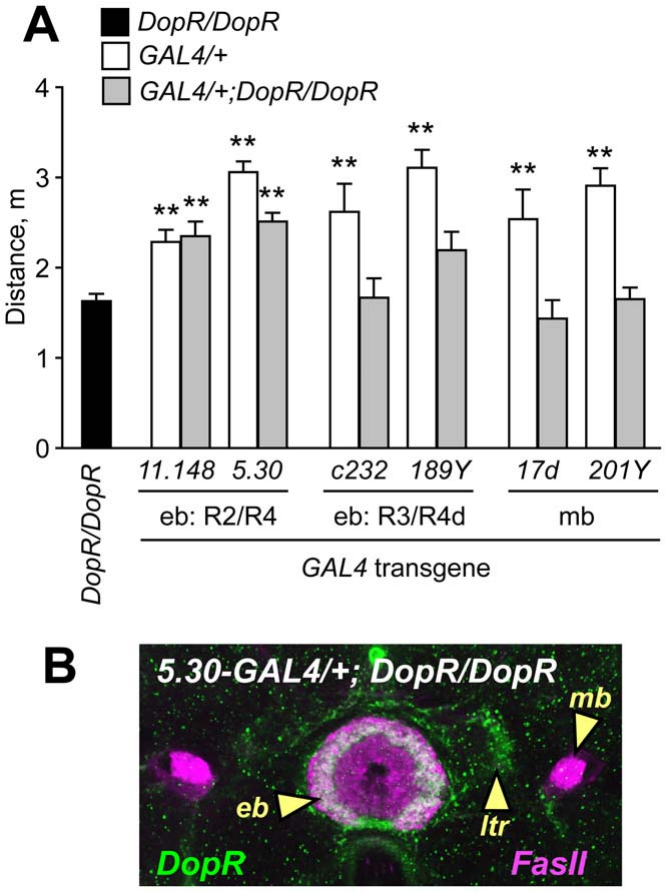
Genetic rescue of *DopR* mutant ethanol-induced hyperactivity by restricted expression of *DopR* in the ellipsoid body. **A**. Distance traveled for animals homozygous for *f02676* (*DopR*/*DopR*), and heterozygous for GAL4 transgenes to drive expression of DopR in patterns that include the ellipsoid body R2/R4 neurons (*11.148*, *5.30*), R3/R4d neurons (*c232*, *189Y*), or the mushroom bodies (*17d*, *201Y*). Restored expression of DopR in the R2/R4 neuron patterns but not others resulted in increased ethanol-induced hyperactivity as compared to *DopR*/*DopR* (**P<0.01, 1 way ANOVA, Dunnett's multiple comparison test, n≥10). **B**. Rescuing strategy results in selectively localized DopR protein expression. Confocal section of an animal homozygous for *f02676* and carrying the GAL4 enhancer trap *5.30*, stained for DopR protein (green) and FasII (magenta). *5.30-GAL4* drives expression of DopR protein in the ring of the ellipsoid body (eb) and the lateral triangles (ltr), but not in the mushroom bodies (mb). DopR expression in the R2 neurons is indicated by the arrowhead.

Our data suggested that DopR may function in the ellipsoid body to promote ethanol-induced hyperactivity. To test this, we carried out genetic rescue experiments, supplying functional DopR to specific brain regions in animals otherwise lacking *DopR*. We took advantage of GAL4-binding sites (*UAS* elements) that are present in the *f02676* transposon. Flies of the genotype *Tub-GAL4/+*; *DopR^f02676^*/+ that express GAL4 ubiquitously from the tubulin promoter showed an almost six-fold increase in *DopR* transcript levels. While the *DopR* transcript produced in these flies is expected to lack sequences encoded by the first exon, resulting in the loss of the predicted translation initiation site (**Fig. 5A**), in-frame methionines encoded in the second exon are likely to initiate translation [21]. Moreover, DopR lacking the long N-terminal extracellular region remains functional in cultured cells [48]. Finally, flies of the genotype *5.30-GAL4/+*; *DopR^f02676^*/*DopR^f02676^* showed selectively restored expression of DopR protein in the ellipsoid body ring and lateral triangles (**Fig. 6B**) indicating that DopR protein was generated and it localized to regions of the ellipsoid body where synaptic connections are likely to be made.

Selectively restoring expression of DopR in *DopR^f02676^* mutants in patterns that included the R2/R4 class ellipsoid body neurons resulted in rescue of ethanol-induced hyperactivity (**Fig. 6A** **, Fig. S7A,B**), indicating that DopR expression in these neurons was sufficient for promotion of hyperactivity by ethanol. Restoring DopR expression in the R3 neurons of the ellipsoid body resulted in a modest but statistically insignificant increase in ethanol-induced hyperactivity with one driver (*189Y*, **Fig. S6C**) but not another (*c232*, **Fig. S7D**). As DopR is expressed broadly in the ellipsoid body (**Fig. 4A,C**), there may be an undetected overlap in expression patterns of the ellipsoid body GAL4 drivers, or there may be differences between the effects of blocking evoked neuronal output and restoring neuronal input. Importantly, restored expression of DopR in the mushroom bodies, where DopR is also normally expressed, did not rescue *DopR^f02676^* ethanol-induced hyperactivity defects (**Fig. 6A** **, Fig. S7E–F**), demonstrating that *DopR* behavioral rescue in the ellipsoid body was specific. Overexpression of *DopR* did not increase ethanol-induced hyperactivity (**Fig. S7G,H**), indicating that the genetic rescue was due to restoration of normal DopR function. Thus, *DopR* expression in the ellipsoid body was sufficient for the promotion of ethanol-induced hyperactivity.

## Discussion

DA regulates specific behavioral responses elicited by acute exposure to drugs of abuse in mammals and in flies. For low to moderate doses of ethanol, DA signaling is associated with euphoria (the subjective high), locomotor activation, and drug seeking. Our studies extend the understanding of the role of DA in ethanol responses in flies in three main ways. First, we identified specific DA (PPM3) and DA target (central complex ellipsoid body) neurons that promote locomotor activation by acute ethanol exposure. Second, we showed that the D1-like receptor DopR is required for promotion of locomotor activity elicited by acute ethanol exposure, and that its function for this role localized to the ellipsoid body. Importantly, DA signaling through DA D1 receptors also promotes locomotor activation by ethanol in mice [6], [49]. Finally, these DA and DA target neurons were dispensable for the olfactory startle response and negative geotaxis, indicating that the circuit regulating locomotor activation by ethanol is separable from at least some other locomotor activation circuits. Our findings allow us to propose a simple DA circuit: ethanol may act either directly or indirectly on PPM3 DA neurons, eliciting a DA locomotor activation signal that is received by DopR in the ellipsoid body, where presumably it is parsed to invoke an appropriate motor response.

### Definition of specific behavioral roles for DA neurons

Silencing of nearly all DA neurons (*TH-GAL4*) or discrete subsets (*Ddc(HL7)-GAL4* and *c346*) resulted in reduced ethanol-induced hyperactivity, whereas silencing of further restricted DA neuron subsets (*Ddc(HL5)-GAL4*) did not. Comparison of expression patterns for *TH*, *Ddc(HL7)* and *Ddc(HL5)* revealed a correlation between effects of TeTx and expression in the PPL1 and PPM3 DA neuron clusters, and a lack of correlation with other DA neuron clusters (**Fig. 1**). Assignment of promotion of ethanol-induced hyperactivity to the PPM3 neurons is based on the phenotypic effects of TeTx silencing of neurons in the *c346* expression pattern, stimulation of locomotion by direct activation of DA neurons in the *c346* pattern, and potentiation of ethanol-induced hyperactivity by overexpression of DAT in either TH or *c346* neurons (**Fig. 2**). Neurons in the PPM3 cluster project to distinct yet highly stereotyped locations in the central complex and are the only DA neurons to target the ellipsoid body and lateral triangles. The *c346* PPM3s project to the ellipsoid body and lateral triangles while other PPM3s project to the fan-shaped body and noduli (**Fig. 2**) [29]. *Ddc(HL7)*, like *c346*, is expressed in a subset of PPM3 neurons (**Fig. S1**), that project to the ellipsoid body (**Fig. S3, Movie S1**). c346-negative PPM3 and the PPL1 neurons may also contribute to ethanol-induced hyperactivity and DA-dependent locomotor activation.

Specific DA-dependent behaviors may map to discrete sets of DA neurons. First, the projection patterns of DA neurons from different clusters are stereotyped and largely non-overlapping [29]. Second, associative olfactory learning is DA-dependent [21], [24], and requires a subset of DA neurons in the PPL1 cluster that project to the mushroom bodies [26]. We did not uncover a DA-dependent role for the mushroom bodies in ethanol-induced hyperactivity (**Fig. 3** **,** **6** **, Fig. S5**), suggesting there exists a separation between DA-dependent locomotor activation and olfactory conditioning. Other DA-influenced behaviors in flies include courtship, arousal state, and a neural correlate of a visual perceptual response [19], [27], [50]. Identifying the DA-dependent neural circuitry for each behavior is likely to help define shared and distinct underlying mechanisms in behavioral control, and will be important for the accurate assessment of gene function in behavior.

What are the inputs and outputs for the PPM3 DA neurons that carry the ethanol locomotor activation signal? Presynaptically localized GFP in the PPM3s labeled the ellipsoid body lateral triangles and ring, suggesting that these are sites of DA release (**Fig. 4L–N**). Morphological analysis of ellipsoid body lateral triangle neurons suggests that this region is dense with dendrites [51], and functional localization of *DopR* to the ellipsoid body for ethanol-induced hyperactivity suggests that this region may be a synaptic target of the PPM3s. The neurons upstream of the PPM3s are not defined, but may include neurons in the central complex or in contact with the lateral processes that extend into the ventral body region (**Fig. 2B,D**). Consistent with this latter possibility, we found that postsynaptically targeted GFP expressed in the PPM3s localized primarily to these lateral processes (not shown). Neurons upstream of the PPM3s could encode information from sensory cues that elicit a locomotor response, including for example the chemical or visual perception of other flies, odors, or food. Alternatively, the PPM3s may function downstream of higher order processing circuits that encode previous experience or interoceptive cues such as satiation state [**52**]. Descriptions of synaptic connectivity outside of the well defined olfactory and visual circuits will be needed to better understand the dopaminergic control of locomotor activity.

### Effects of DAT manipulation on ethanol behavioral responses

Reduced and increased DAT expression resulted in pronounced decreases and increases in ethanol-induced hyperactivity, respectively, helping to confirm that DA regulates ethanol-induced hyperactivity (**Fig. S1A**, **2K**). The effect of loss of DAT on DA neurotransmission is likely to be quite complex, and may include increased dopamine tone, recruitment of extrasynaptic DA targets, depletion of readily releasable pools of DA, and compensatory changes in other dopamine signaling molecules. Similarly, increased DAT levels may affect any of these parameters. Acute ethanol exposure increases dopamine release in the nucleus accumbens that is accompanied by increased locomotor activity in mice [53]. To our knowledge, the effects of DAT knockout on ethanol stimulation of locomotion in mice is not known. However, a model in flies whereby ethanol-evoked release of dopamine in DAT mutants results in higher-than-normal extracellular DA levels does not fit in a simple manner with the reduced hyperactivity of DAT mutants seen in our experiments. A recent report showed that reuptake by the serotonin transporter was crucial for maintaining serotonin releasable pools in *Drosophila*, raising the possibility that a similar mechanism functions at dopaminergic synapses in flies [54]. Our finding that the maintenance of increased locomotor activity required continuous activation of TH neurons is consistent with this interpretation (**Fig. 2H**).

### Role of the ellipsoid body in control of motor behavior and ethanol responses

The ellipsoid body is a prominent central neuropil that has been implicated in regulating unstimulated locomotor activity levels [55], the visual control of walking and flight [56]–[58], ethanol tolerance [37], and a persistent state of arousal induced by repeated mechanical stimulation [27]. Current findings suggest that specific motor behaviors may map to distinct neuronal classes within the ellipsoid body; for example, orientation during visually guided walking maps to the R1 or R3 neurons [57], [59], whereas ethanol-induced hyperactivity maps to the R2/R4 neurons (**Fig. 3** **,** **6**). While the specific inputs and outputs of the ellipsoid body are not yet known (with the exception of the DA input described here), the above findings, taken together with earlier structural and lesioning studies, have led to the hypothesis that the ellipsoid body may be involved in parsing information from various sensory modalities to engage the appropriate motor output [36]. Interestingly, DopR in the R2/R4 neurons also regulates arousal state [27]. However, while ellipsoid body-expressed DopR promotes locomotor activity induced by ethanol, it suppresses locomotor activity induced by repeated mechanical stimulation. Although alternative interpretations exist, these apparently opposite roles for DopR may suggest the integration of other sources of information to set the sign of dopamine signaling in the ellipsoid body with respect to motor output. Conceptually consistent with this idea, a previous study showed that DA neuron stimulation in low activity flies increased activity, whereas high activity flies showed the opposite response [60]. Additionally, the contribution to fly locomotor behaviors of other D1-class (*DopR2*/*DAMB/DopR99B*, *DmDopEcR*) and D2-class (*D2R*) dopamine receptors is largely unknown [61]–[64]. Pharmacological and gene knockout studies in rodents indicate that both D1-class and D2-class DA receptors regulate the locomotor stimulant effects of ethanol [65], [66].

The R2/R4 neurons of the ellipsoid body also regulate ethanol sedation sensitivity and sedation tolerance via a Homer protein-dependent mechanism [37]. Therefore, the ellipsoid body R2/R4 neurons regulate ethanol-induced locomotor activity in a DA-dependent fashion, and ethanol sedation sensitivity and tolerance in a Homer-dependent fashion. In mammals, DA and glutamate are thought to play distinct a roles in the nucleus accumbens for the behavioral effects of ethanol [67]. Dopaminergic neurons from the ventral tegmental area and glutamatergic neurons from cortical and other brain regions make synapses onto GABAergic medium spiny neurons in the nucleus accumbens where they regulate ethanol-induced locomotor activity (DA) and ethanol consumption and withdrawal (glutamate) [68]. It is of note that there are both GABAergic and glutamatergic neurons in the ellipsoid body [69], [70], and that a subset of R2/R4 neurons in the *5.30* and *11.148* GAL4 expression patterns are GABAergic (not shown). Thus, *Drosophila* may serve as a model to define some fundamental properties of neural circuit function as well as gene action that may be relevant to the actions of drugs of abuse in mammals.

### Locomotor activation and reward

Selective breeding studies in rodents uncovered a positive correlation between the locomotor stimulant and reinforcing properties of ethanol [2], [3], and both properties of ethanol are mediated at least in part through the mesolimbic DA pathway [71], [72]. Moreover, human studies demonstrated a correlation between ethanol sensitivity and the propensity to develop alcohol use disorders [1]. The mesolimbic DA-dependent acute locomotor and reinforcing effects of cocaine, however, are genetically separable [73], [74], suggesting that DA can encode the acute and reward learning effects of at least some drugs of abuse in a complex manner. Our current findings show that there exists a DA circuit that mediates the locomotor stimulant properties of ethanol in flies. Application of learned reward-like behavior models for drugs of abuse and other stimuli to DA circuit analysis in flies will aid in understanding how this organism codes for complex DA-dependent behaviors.

Because DopR regulates arousal levels in a set of ellipsoid body neurons that overlap with those required for ethanol-induced hyperactivity [27], it is possible that ethanol-stimulated locomotion represents a form of arousal in flies. A proposed function for mesolimbic DA in mammals is to impart salience to increase the motivational state with regard to cues in the environment [4]. It will be important to determine whether locomotor activating stimuli other than ethanol also utilize this circuit, and to determine how ethanol interacts with other locomotor-activating cues that elicit approach and avoidance responses.

## Materials and Methods

### Genetics and Molecular Biology

All strains were outcrossed to the wild-type Berlin strain carrying the *white* (*w^1118^*) mutation (control) for 5–10 generations. *DAT^fmn^* was followed molecularly during outcross by PCR with the upstream primer 5′-GCTGCTGGCCTATGCATCC and the downstream primers 5′-GCAATGCACCCATGTCGCC in the *fmn* lesion and 5′-ACTGGTTAACAAAGCATCC downstream of the *fmn* lesion. *DopR* locus lesions included *f02676* (*PBac*{WH}), *PL00420* (*PBac*{GAL4D,EYFP}), *dumb^1^* (*In(3LR)234*) [21], and *Df(3R)ED5634*. Transposon marker genes are *mini-w^+^* for *f02676* and *EYFP* for *PL00420*. Some strains (*2.70*, *3.16*, *4.13*, *4.67*, *4.69*, *5.21*, *5.30*, *5.36*, *6.61*, *11.17*, *11.32*, *11.33*, *11.113*, *11.148*, *12.30*) were generated by mobilization of the GAL4-containing enhancer trap transposon *P*{GawB} to random integration sites in the genome. *UAS-CD2-mCherry* was a gift from Bing Ye, *UAS-tdTomato-GFP-HIP14* was from Steve Stowers, and *UAS-dTrpA1* was from Paul Garrity. *TH-GAL4*, *UAS-TeTx* (*UAS-TeTxLC.tnt*), *UAS-IMP* (*UAS-TeTxLC.IMPTNT*), and *UAS-n-syb-eGFP* were obtained from the Bloomington stock center. The *P*{GawB} strains *78Y*, *189Y*, *c42*, *c107*, *c161*, *c232*, *c346*, *c561*, and *c819* were obtained from the Fly-trap project (www.fly-trap.org). To visualize individual neurons in the *TH-GAL4* pattern, flies heterozygous for *TH-GAL4*, *hs-Flp*, and *UAS >CD2*, *y+ >CD8-GFP* were heat shocked at 37°C for 10–30 min during the first larval instar. Over 500 brains were analyzed. The transposon *f02676* was excised utilizing standard genetic methods and was backcrossed for ten generations to *f02676*. DopR antisera was raised in rabbits against the peptide sequence CVAEKQRYKSIRRPKNQPKKFK derived from the intercellular loop region between transmembrane regions five and six, and affinity purified using the peptide (Covance, Denver, PA). Antisera specificity was verified by ELISA and immunostaining of *DopR^f02676^* brains. The *DopR* open reading frame was isolated by PCR from the Berlin strain with the primers 5′-ATGTACACACCACACCCA and 5′-TCAAATCGCAGACACCTGC and cloned into pUAST to generate *UAS-DopR*.

Quantitative PCR was carried out according to the manufacturer's instructions (Applied Biosystems) on an ABI PRISM 7900 Sequence Detection System, using expression levels of *RpL32* as a standard to normalize sample concentrations. The relative transcript expression levels were determined by comparison of mutant strains to the genetic background control. Taqman probesets (Applied Biosystems) used in this study were *D2R*: Dm01845573_g1, *DopR*: Dm02134814_m1, *DopR2*: Dm02151743_g1, and *RpL32*: Dm02151827_g1.

### Construction of Ddc/GAL4 fusion genes

Numbering is relative to the *Ddc* transcription start site [75], and underlined letters represent *Ddc* exons. See **Fig. S1C** for diagrams of the constructs.

#### HL5-[DdcA/GAL4]

A fragment of *Ddc* from Bgl II at −2702 through a PCR-added BamHI site at +160 in exon A, was cloned upstream of *GAL4* at a BamHI site of the pGaTB vector [76] to generate construct HL5 [DdcA/Gal4]. The 190 bp portion (−24 to +160) downstream of this *Ddc* fragment was a PCR product and isolated by digestion with NcoI and BamHI. The PCR fragment was generated using oligonucleotides: 5′-CGGTCCTGCGGAATTGGCAGCGCTGC sense primer for *Ddc* 5′ flanking : −67→−42, and 5′-CGCGGATCCGCGCACTTGTTGCCG antisense primer in *Ddc* exon A, adding BamHI at +158→146.

#### HL7-[DdcA/Gal4/DdcABCD]

To generate [DdcA/Gal4/DdcABC], *Ddc* sequences from exon A through exon D were subcloned downstream of the hsp70 polyA sequences in HL5. The *Ddc* HpaI fragment was isolated by restriction digestion of HpaI sites at +175 in exon A and at +3756 in exon D and, ligated downstream of hsp70 of the HL5 SpeI site. The SpeI site was immediately downstream of the Ddc/GAL4 insert. The 5′ protruding SpeI ends were blunted in order to ligate with the *Ddc* HpaI fragment. The [DdcAB/Gal4/Ddc3'end] and [DdcA/Gal4/DdcABCD] fragments in HL7 were subcloned into P-element transformation vector CaSpeR4 at the KpnI and SpeI sites, respectively.

The constructs in P-element CaSpeR4 vectors were purified using maxi-purification method (Qiagen Plasmid Kit). Transgenic lines were generated by microinjection of purified DNA into *w^1118^* embryos [77], [78].

### Immunohistochemistry

Brains dissected from 2–5 day old adult flies were fixed for 16 hr at 4°C in 2% paraformaldehyde in PBT (0.05% Triton X-100 in phosphate buffered saline). Primary and secondary antibodies were incubated with fixed tissue at 4°C for 48 hr each in 0.5% BSA and 5% normal goat serum in PBT, with extensive washes with PBT between steps. Images were collected on a Zeiss confocal microscope after mounting brains in Vectashield (Vector Laboratories). Confocal stacks were manipulated in ImageJ and Photoshop. Antibodies used were MAb 1D4 anti-FasII (1∶40, Developmental Studies Hybridoma Bank, Iowa), rabbit anti-GFP (1∶1000, Invitrogen), mouse anti-GFP (1∶500, Zymed), rabbit anti-TH (1∶100, Pel-Freez), and rabbit anti-DopR (1∶1250).

### Behavioral Analysis

Flies were maintained on a standard cornmeal/molasses/yeast media at 25°C and 70% humidity with an approximately 16hr/8hr light/dark schedule. For behavioral testing, 25 males and virgin females were mated in bottles with 50 mL standard media with a few added grains of Baker's yeast for 2 days, and groups of 23 male progeny were collected into standard food vials without yeast 11 days later. “*n*” indicates the number of groups of flies of a given genotype derived from independent parental crosses that were tested on separate days. Flies were allowed to recover from CO_2_ anesthesia for 2 days, and were tested behaviorally between 11 am and 6 pm. All behavioral tests, unless noted otherwise, were carried out at 25°C with constant illumination, utilizing the booz-o-mat, an eight-chambered manifold holding 16×125 mm cylindrical tubes [11]. Ethanol vapor was delivered to the booz-o-mat as a continuous stream by bubbling air separately through a 3L flask of 95% ethanol or water. Ethanol and humidified air streams were mixed at different ratios to a final flow rate of 5.5 L/min. Locomotor tracking analysis was as described previously [11]. Ethanol-induced hyperactivity was quantified as the area under the curve from 2–12.5 min exposure for 47% and from 2–10 min exposure for 60% ethanol vapor, corresponding to the time of hyperactivity onset to peak speed achieved for control flies at each concentration [13]. Hyperactivity was also quantified as the locomotor speed achieved at 7.5 min of ethanol exposure (**Table S1**). Unstimulated locomotor activity was measured in 60×90×10 mm chambers in constant light in the absence of food or humidified air, and was quantified as the area under the curve from 10–60 min. For TrpA1 activation tests, 10–20 male flies were placed in a thin-walled 30×50×2 mm Plexiglas chamber on the surface of a 96-well thermal cycler heat block (MJ Research) and were allowed to acclimate for 10 min at 15°C. They were filmed for 5 min at 15°C, 45 min at 30°C, and 5 min at 15°C. Locomotor activity was averaged over 15 sec every 2 min, and the distance traveled from 20–30 min at 30°C was calculated for statistical analysis. Statistical analyses were carried out in Minitab v.15 and Prism 4. Error bars in all figures indicate SEM.

### Ethanol sedation kinetics and absorption

Groups of twenty genetically identical 2–4 day old adult male flies were exposed to 60% ethanol vapor in the booz-o-mat. At 5 min intervals the flies were agitated by rotating the tubes, and the number of flies that were unable to right themselves following a 10 sec recovery were counted. The average of 10 experiments from 5 days was determined. For ethanol tolerance, the number of flies sedated following 26 min exposure, approximately the time to 50% sedation for control flies, were counted. Ethanol absorption was measured by exposing groups of 25 flies to either ethanol vapor (47%) or humidified air for 30 min. Flies were immediately frozen on dry ice and the ethanol concentration in whole fly homogenates was measured with an alcohol dehydrogenase-based spectrophotometric assay (Diagnostic Chemicals, Ltd).

## Supporting Information

Table S1 Locomotor speed achieved at 7.5 min ethanol exposure.
(0.06 MB PDF)

Figure S1 Regulation of ethanol-induced hyperactivity by dopamine (DA). **A1**. Reduced ethanol-induced hyperactivity in flies homozygous for the DAT DA transporter mutation *fmn*. **A2**. Distance traveled (2–12.5 min) is reduced in *DAT^fmn^* (**P<0.0001, 2 sample t-test, n = 12). *DAT^fmn^* was recessive for this phenotype. **B**. Pharmacological reduction of DA in adult flies reduced ethanol-induced hyperactivity. **B1**. Wild-type control (*w*- Berlin) flies were fed 10 mg/mL 3-iodotyrosine in 2% yeast/5% sucrose for 36–48 hr, and then exposed to 45% ethanol vapor. **B2**. Drug fed flies showed a reduction in distance traveled (**P = 0.0072, paired t-test. n = 10). **C**. Diagram depicting the *Ddc-GAL4* transgenes used in this study. See Methods for construction details. **D**, **F**. Expression of *Ddc(HL7)-GAL4* and *Ddc(HL5)-GAL4* in TH-positive neurons. Posterior groups of TH-positive neurons (magenta) and GAL4-positive neurons expressing *UAS-GFP* (green) are shown. **E**, **G**. Locomotor activity profiles of flies expressing either TeTx or IMP in the *Ddc(HL7)* (E) and *Ddc(HL5)* (G) patterns and exposed to 47% ethanol vapor.
(7.19 MB TIF)

Figure S2 Role of the thoracic ganglion in ethanol-induced hyperactivity. **A–D**. Expression pattern of the indicated GAL4 transgenes (detected with *UAS-GFP*, green) in the adult thoracic ganglion, counterstained with TH antibodies (magenta). **A**. *Ddc(HL5)-GAL4* is expressed in most TH-positive neurons in the thoracic ganglion. Positions of TH-positive cell bodies are indicated by arrowheads. No overlap was evident of GAL4 drivers *c346*, *5.30*, and *11.148* with TH expression in the thoracic ganglion. **E**. Ethanol-induced locomotor activity (0–25 min) in freshly decapitated and matched unoperated controls. ‘A’ indicates locomotor speed in humidified air 1 min prior to ethanol exposure. The righting response and grooming activity were intact in decapitated flies just prior to ethanol exposure, indicating that the preparation was behaviorally responsive. n = 3.
(9.47 MB TIF)

Figure S3 Expression of *Ddc(HL7)-GAL4* and *Ddc(HL5)-GAL4* (detected by *UAS-GFP*, green) in the ellipsoid body ring and lateral triangles, counterstained with TH antibodies (magenta).
(5.94 MB TIF)

Figure S4 **A–F**. Locomotor activity profiles for the functional mapping of ellipsoid body neurons in ethanol-induced hyperactivity. Flies of the indicated genotypes were exposed to 47% ethanol vapor (open box on horizontal axis). GAL4 lines are indicated on each graph. **G**,**H**. Expression of the indicated GAL4 lines in R2/R4 neurons of the ellipsoid body (arrowhead), detected by GFP expression and counterstained with anti-Bruchpilot (nc82) to highlight the synaptic neuropil. **I**. Higher resolution analysis of startle magnitude for the GAL4 lines expressed in the R2/R4 neurons, corrected for pre-exposure locomotor activity levels. For *5.30* and *11.148*, the olfactory startle responses were unaffected by TeTx expression (*5.30*: P = 0.4403, *11.148*: P = 0.0556, 1 way ANOVA, n≥5). For *c819*, *c819/+*;*UAS-IMP/+* was reduced (P = 0.0194, 1 way ANOVA, Tukey's multiple comparison test, n≥3).
(5.76 MB TIF)

Figure S5 Mushroom body ablation. **A**. Ethanol-induced hyperactivity at 60% ethanol vapor with (+HU) and without (-HU) hydroxyurea treatment. Distance traveled 2–12.5 min did not differ (P = 0.0861, paired t-test, n = 4). **B**, **C**. Brains from control and HU treated flies, stained with anti-FasII to highlight the mushroom bodies (arrowheads) and the central complex ellipsoid body (arrow), which is unaffected by HU treatment.
(1.33 MB TIF)

Figure S6 *DopR* mutant behavioral characterization. **A**. Ethanol-induced sedation was unaffected in *f02676* homozygotes, measured as the loss of the ability to right. (60% ethanol vapor, P = 0.0945, 2 sample t-test, n = 10). **B**. Unstimulated locomotor activity, measured for 60 min immediately after flies were introduced into a 60×90×10mm Plexiglas box. *DopR^f02676^* showed higher activity (distance traveled 10–60 min, P = 0.0009, 2 sample t-test, n = 10). **C**, **D**. Precise excision of *f02676* reverts ethanol-induced hyperactivity and unstimulated activity behavioral phenotypes. **C**. Ethanol-induced hyperactivity for the genetic background control, *f02676* homozygotes, and two independent precise excisions of *f02676* (*Ex1* and *Ex2*). **D**. Unstimulated activity for the same strains. Distance traveled from 10–60 min showed that *f02676* is different from all other genotypes (P<0.001, 1 way ANOVA, Tukey's multiple comparison test, n = 5). **E**. Ethanol-induced hyperactivity in animals homozygous for *PL00420*. **F**. Unstimulated activity was unaffected in *PL00420* homozygotes. **G**. Non-complementation for ethanol-induced hyperactivity by *DfED5634*, a deficiency that deletes the entire *DopR* locus, for *f02676* and the *dumb^1^* inversion allele.
(0.65 MB TIF)

Figure S7 **A–F**. Locomotor activity profiles for the genetic rescue of the *DopR^f02676^* mutant ethanol-induced hyperactivity phenotype. **G**. Expression of *DopR* utilizing the *UAS* sites in *f02676* or a *UAS-DopR* transgene has no effect on ethanol-induced hyperactivity in flies that are either wild-type or heterozygous for *DopR* (left group: P = 0.7842, 1 way ANOVA, n = 8, right group: P = 0.575, 1 way ANOVA, n = 5). **H**. Increased *DopR* transcript levels when *UAS-DopR* is combined with the pan-neuronal *elav-GAL4* (**P = 0.0062, 1 way ANOVA, Tukey's multiple comparison test, n = 3).
(0.79 MB TIF)

Movie S1 PPM3 neurons labeled by c346, detected by UAS-CD8-GFP, confocal stack reconstruction.
(0.92 MB MOV)
