# Supplementary material for: A Pair of Dopamine Neurons Target the D1-Like Dopamine Receptor DopR in the Central Complex to Promote Ethanol-Stimulated Locomotion in Drosophila
Source: PLoS One. 2010 Apr 1;5(4):e9954. doi: 10.1371/journal.pone.0009954 (PMC2848596; doi:10.1371/journal.pone.0009954)
Supplement: Table S1 — Locomotor speed achieved at 7.5 min ethanol exposure. (0.06 MB PDF) [file pone.0009954.s001.pdf]

Experiment	Fig.	Genotype or Condition	Speed, mm/sec (SEM)	Statistical Test	Test Result	Post-hoc	Post-hoc Results
Tetanus in *TH-GAL4* neurons	1C	*TH-GAL4/+; UAS-TeTx/+*	1.80 (0.190)	1-way RM ANOVA	P<0.0005	Dunnett	*vs. TH-GAL4/+; UAS-TeTx/+:*
		TH-GAL4/+; UAS-IMP/+	3.55 (0.405)				P<0.01
		TH-GAL4/+	2.76 (0.324)				P<0.01
		UAS-TeTx/+	3.59 (0.349)				P<0.01
		UAS-IMP/+	2.72 (0.265)				P<0.01
Tetanus in *Ddc(HL7)-GAL4* neurons	1C	*Ddc(HL7)-GAL4/+; UAS-TeTx/+*	2.39 (0.312)	2-sample t-test	P=0.0026		
		Ddc(HL7)-GAL4/+; UAS-IMP/+	4.34 (0.385)				
Tetanus in *Ddc(HL5)-GAL4* neurons	1C	*Ddc(HL5)-GAL4/+; UAS-TeTx/+*	5.79 (0.826)	2-sample t-test	P=0.450		
		Ddc(HL5)-GAL4/+; UAS-IMP/+	6.54 (0.320)				
DAT^{fmn} hyperactivity	SF1A	*fmn/fmn*	1.97 (0.096)	Paired t-test	P<0.0005		
		Control	3.67 (0.228)				
3IY treatment	SF1C	3IY	3.07 (0.182)	Paired t-test	P=0.0234		
		Control	3.61 (0.309)				
Tetanus in *c346* neurons	2F	*c346/Y; UAS-TeTx/+*	5.20 (0.900)	1-way ANOVA	P=0.0026	Tukey	P<0.01 *c346/TeTx vs. c346/IMP*
		c346/Y; UAS-IMP/+	8.88 (0.407)				P<0.05 *c346/TeTx vs. c346/Y*
		c346/Y	8.52 (0.534)				P>0.05 *c346/IMP vs. c346/Y*
DAT overexpression	2J,K	*TH-GAL4/+; UAS-DAT/+*	4.22 (0.235)	2-sample t-test	P<0.0005		
		TH-GAL4/+	2.59 (0.095)				
DAT overexpression	2J,K	*c346/Y; UAS-DAT/+*	5.77 (0.203)	2-sample t-test	P=0.0013		
		c346/Y	4.46 (0.293)				
Tetanus in Central Complex	3A	*5.30/+; UAS-TeTx/+*	3.14 (0.315)	1-way ANOVA	P=0.0008	Tukey	P<0.001 *5.30/TeTx vs 5.30/IMP*
		5.30/+; UAS-IMP/+	6.85 (0.831)				P<0.01 *5.30/TeTx vs. 5.30/+*
		5.30/+	5.94 (0.481)				P>0.05 *5.30/IMP vs. 5.30/+*
		11.148/+; UAS-TeTx/+	3.70 (0.182)	1-way ANOVA	P=0.0051	Tukey	P<0.05 *11.148/TeTx vs. 11.148/IMP*
		11.148/+; UAS-IMP/+	6.00 (0.679)				P<0.05 *11.148/TeTx vs. 11.148/+*
		11.148/+	5.86 (0.726)				P=0.136 *11.148/IMP vs. 11.148/+*
		c819/+; UAS-TeTx/+	6.92 (0.521)	1-way ANOVA	P=0.0024	Tukey	P<0.05 *c819/TeTx vs. c819/IMP*
		c819/+; UAS-IMP/+	8.72 (0.279)				P<0.01 *c819/TeTx vs. c819/+*
		c819/+	10.40 (0.750)				P>0.05 *c819/IMP vs. c819/+*
		189Y/+; UAS-TeTx/+	8.97 (0.325)	1-way ANOVA	P=0.0337	Tukey	P>0.05 *189Y/TeTx vs. 189Y/IMP*
		189Y/+; UAS-IMP/+	8.45 (0.510)				P<0.05 *189Y/TeTx vs. 189Y/+*
		189Y/+	7.10 (0.631)				P<0.05 *189Y/IMP vs. 189Y/+*
		c561/Y; UAS-TeTx/+	8.14 (0.508)	1-way ANOVA	P=0.0448	Tukey	
		c561/Y; UAS-IMP/+	8.59 (0.637)				P>0.05 for all comparisons
		c561/Y	7.12 (0.245)				
		c232/+; UAS-TeTx/+	8.44 (0.766)	1-way ANOVA	P=0.9939		
		c232/+; UAS-IMP/+	8.55 (0.450)				
		c232/+	8.39 (1.330)				
$DopR^{f02676}$ hyperactivity	5D	*f02676/f02676*	2.28 (0.227)	1-way ANOVA	P=0.0014	Tukey	P<0.001 *f02676/f02676* vs. +/+
		f02676/+	2.99 (0.221)				P>0.05 for others
		+/+	3.52 (0.207)				
f02676 Excision	5E	*f02676/f02676*	2.65 (0.232)	1-way RM ANOVA	P=0.0024	Dunnett	*vs. f02676/f02676:*
		Excision 1	3.49 (0.226)				P<0.01
		Excision 2	3.48 (0.197)				P<0.05
		Control	3.67 (0.228)				P<0.01
DopR(PL00420) hyperactivity	5F	*PL00420/PL00420*	2.18 (0.222)	2-sample t-test	P=0.0368		
		Control	2.94 (0.262)				
DopR allelic complementation	5G	*DfED5634/+*	5.67 (0.302)	1-way ANOVA	P<0.0005	Dunnett	*vs. DfED5634:*
		DfED5634/f02676	2.60 (0.205)				P<0.01
		DfED5634/dumb1	3.03 (0.514)				P<0.01
DopR Rescue	6A	*f02676/f02676*	2.67 (0.140)	1-way ANOVA	P<0.0005	Dunnett	*vs. f02676/f02676:*
		11.148/+	3.81 (0.260)				P<0.05
		11.148/+; f02676/f02676	3.90 (0.341)				P<0.05
		5.30/+	5.37 (0.230)				P<0.01
		5.30/+; f02676/f02676	4.14 (0.189)				P<0.01
		c232/+	4.51 (0.596)				P<0.01
		c232/+; f02676/f02676	2.64 (0.403)				P>0.05
		189Y/+	5.48 (0.408)				P<0.01
		189Y/+; f02676/f02676	3.58 (0.376)				P>0.05
		17d/+	4.20 (0.637)				P<0.05
		17d/+; f02676/f02676	2.29 (0.355)				P>0.05
		201Y/+	5.40 (0.418)				P<0.01
		201Y/+; f02676/f02676	2.64 (0.240)				P>0.05
DopR overexpression	SF7G	*11.148/+; f02676/+*	2.99 (0.570)	1-way RM ANOVA	P=0.0903		
		11.148/+	3.04 (0.398)				
		f02676/+	1.95 (0.176)				
DopR overexpression	SF7G	*elav-GAL4(3E1)/+; UAS-DopR/+*	3.76 (0.618)	1-way RM ANOVA	P=0.2416		
		elav-GAL4(3E1)/+	3.70 (0.340)				
		UAS-DopR/+	3.20 (0.553)				
