# Supplementary figures and images for: A Pair of Dopamine Neurons Target the D1-Like Dopamine Receptor DopR in the Central Complex to Promote Ethanol-Stimulated Locomotion in Drosophila
Source: PLoS One. 2010 Apr 1;5(4):e9954. doi: 10.1371/journal.pone.0009954 (PMC2848596; doi:10.1371/journal.pone.0009954)

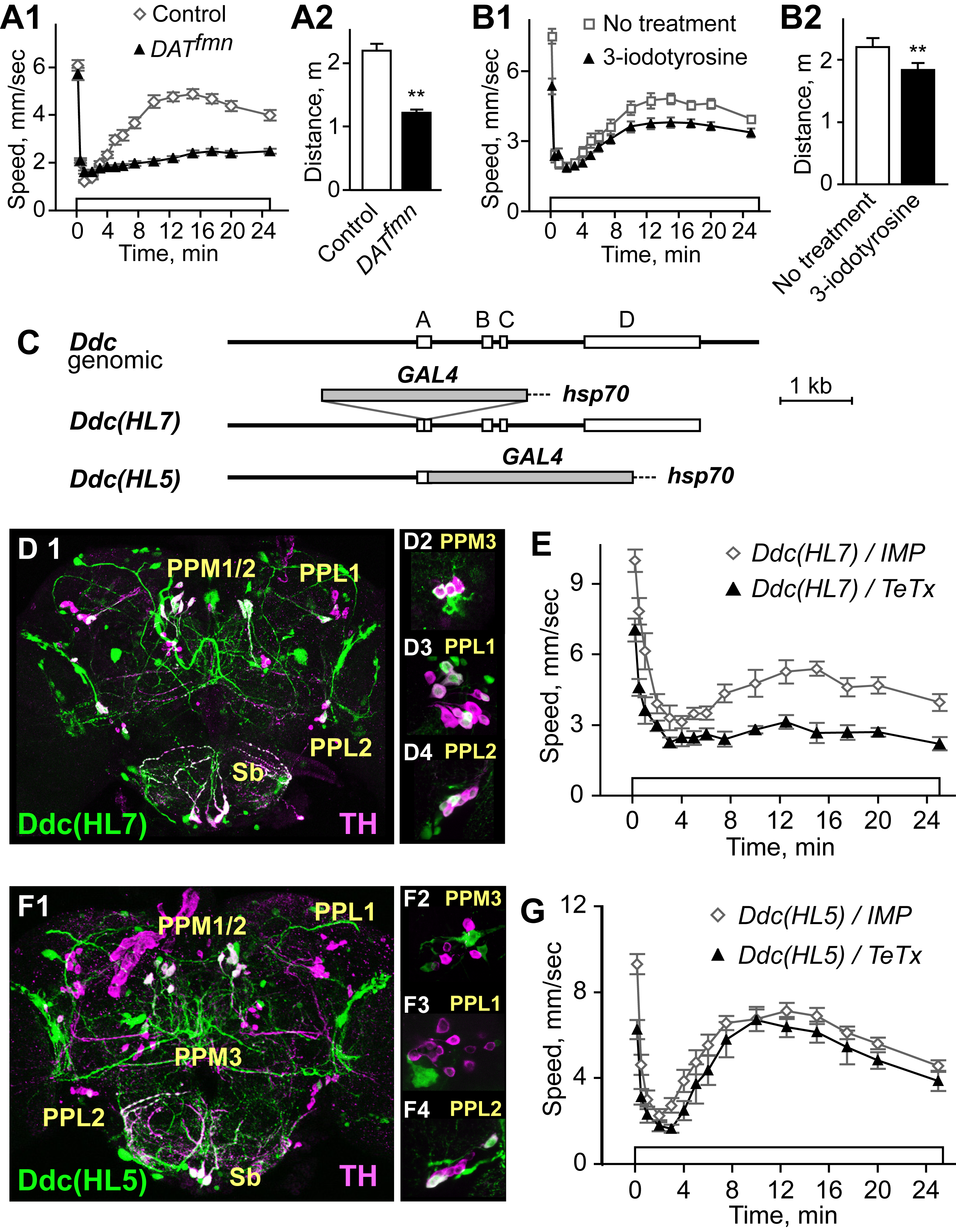

Supplement: Figure S1 — Regulation of ethanol-induced hyperactivity by dopamine (DA). A1. Reduced ethanol-induced hyperactivity in flies homozygous for the DAT DA transporter mutation fmn. A2. Distance traveled (2–12.5 min) is reduced in DATfmn (**P<0.0001, 2 sample t-test, n = 12). DATfmn was recessive for this phenotype. B. Pharmacological reduction of DA in adult flies reduced ethanol-induced hyperactivity. B1. Wild-type control (w- Berlin) flies were fed 10 mg/mL 3-iodotyrosine in 2% yeast/5% sucrose for 36–48 hr, and then exposed to 45% ethanol vapor. B2. Drug fed flies showed a reduction in distance traveled (**P = 0.0072, paired t-test. n = 10). C. Diagram depicting the Ddc-GAL4 transgenes used in this study. See Methods for construction details. D, F. Expression of Ddc(HL7)-GAL4 and Ddc(HL5)-GAL4 in TH-positive neurons. Posterior groups of TH-positive neurons (magenta) and GAL4-positive neurons expressing UAS-GFP (green) are shown. E, G. Locomotor activity profiles of flies expressing either TeTx or IMP in the Ddc(HL7) (E) and Ddc(HL5) (G) patterns and exposed to 47% ethanol vapor. (7.19 MB TIF) [file pone.0009954.s002.tif]

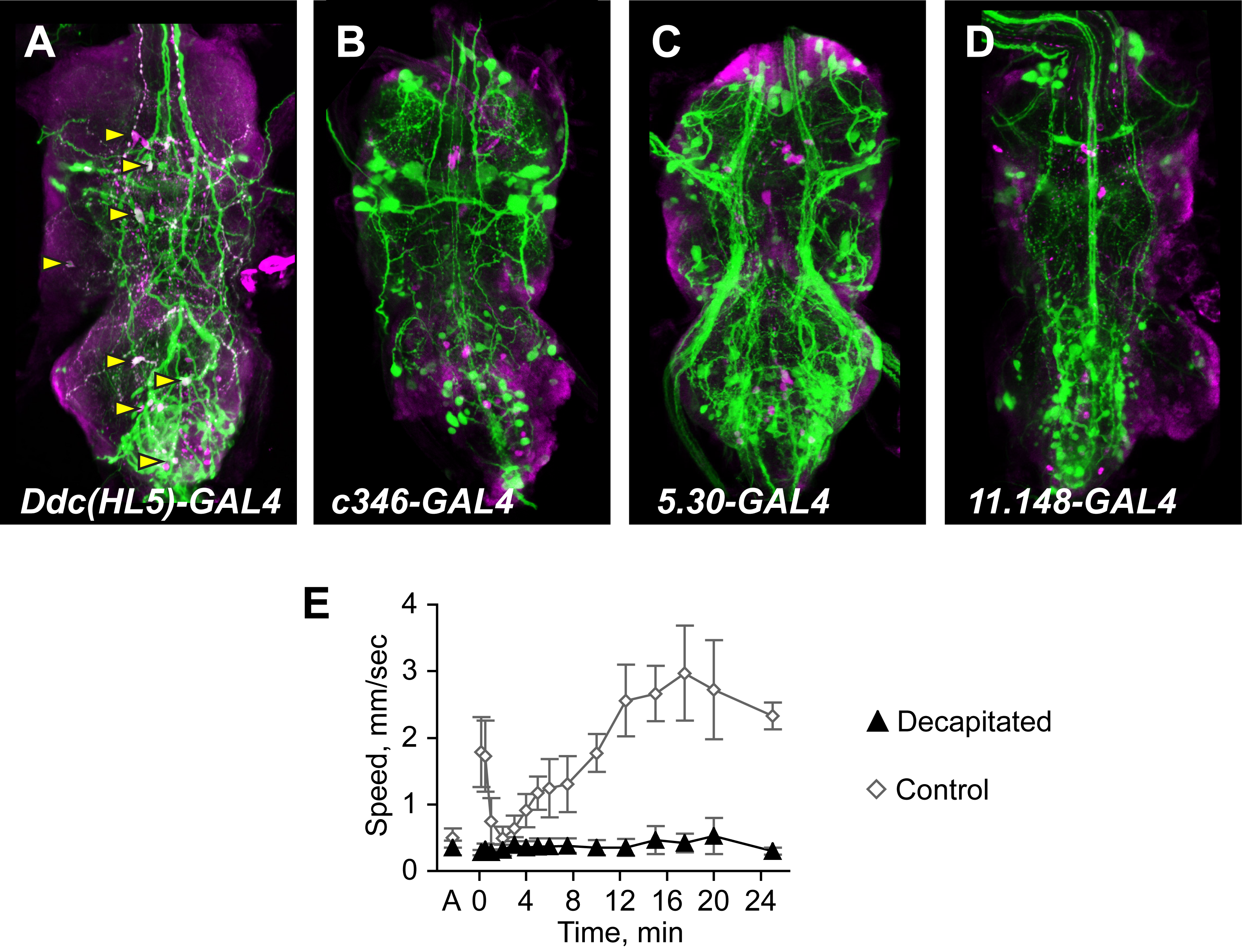

Supplement: Figure S2 — Role of the thoracic ganglion in ethanol-induced hyperactivity. A–D. Expression pattern of the indicated GAL4 transgenes (detected with UAS-GFP, green) in the adult thoracic ganglion, counterstained with TH antibodies (magenta). A. Ddc(HL5)-GAL4 is expressed in most TH-positive neurons in the thoracic ganglion. Positions of TH-positive cell bodies are indicated by arrowheads. No overlap was evident of GAL4 drivers c346, 5.30, and 11.148 with TH expression in the thoracic ganglion. E. Ethanol-induced locomotor activity (0–25 min) in freshly decapitated and matched unoperated controls. ‘A’ indicates locomotor speed in humidified air 1 min prior to ethanol exposure. The righting response and grooming activity were intact in decapitated flies just prior to ethanol exposure, indicating that the preparation was behaviorally responsive. n = 3. (9.47 MB TIF) [file pone.0009954.s003.tif]

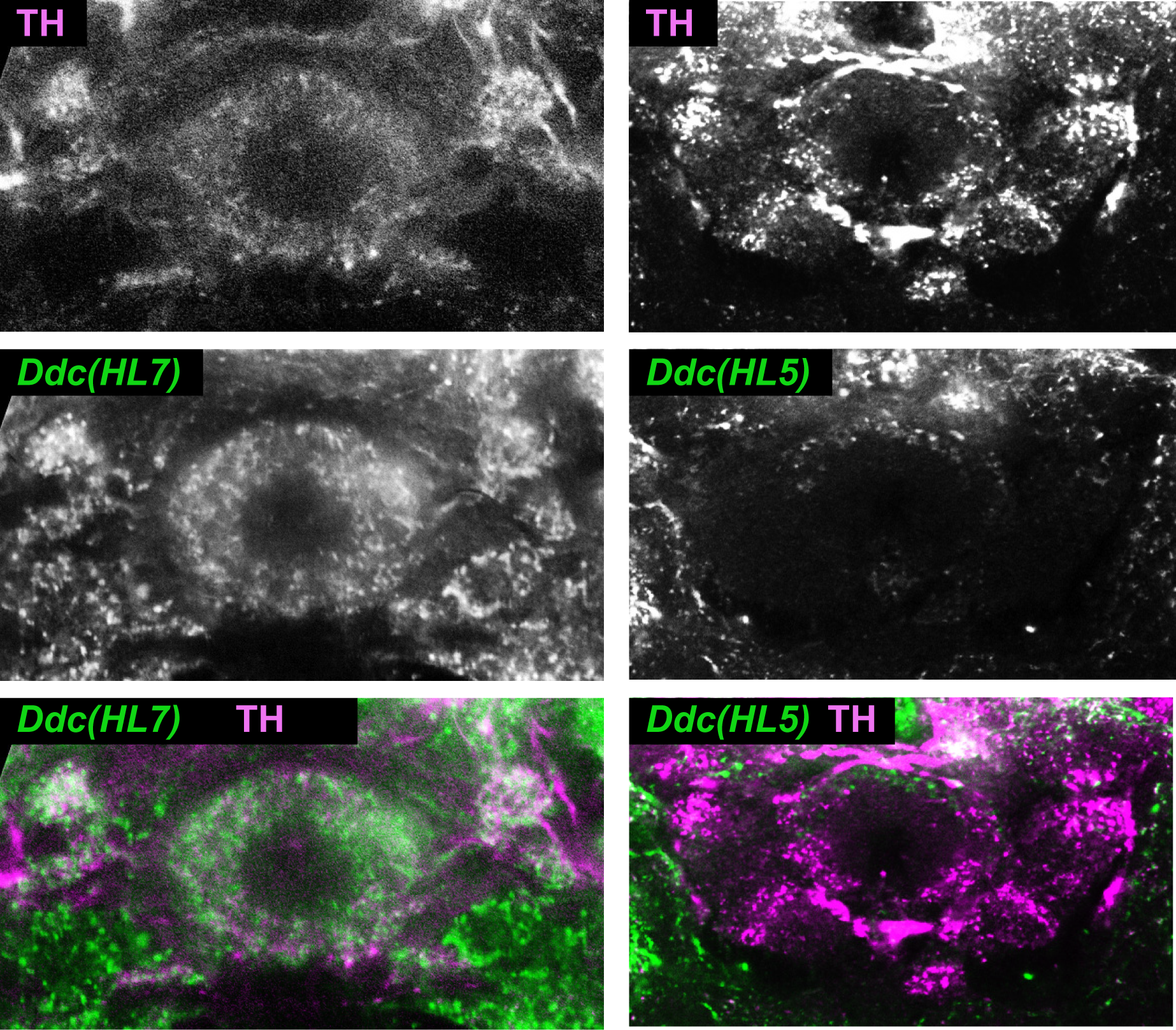

Supplement: Figure S3 — Expression of Ddc(HL7)-GAL4 and Ddc(HL5)-GAL4 (detected by UAS-GFP, green) in the ellipsoid body ring and lateral triangles, counterstained with TH antibodies (magenta). (5.94 MB TIF) [file pone.0009954.s004.tif]

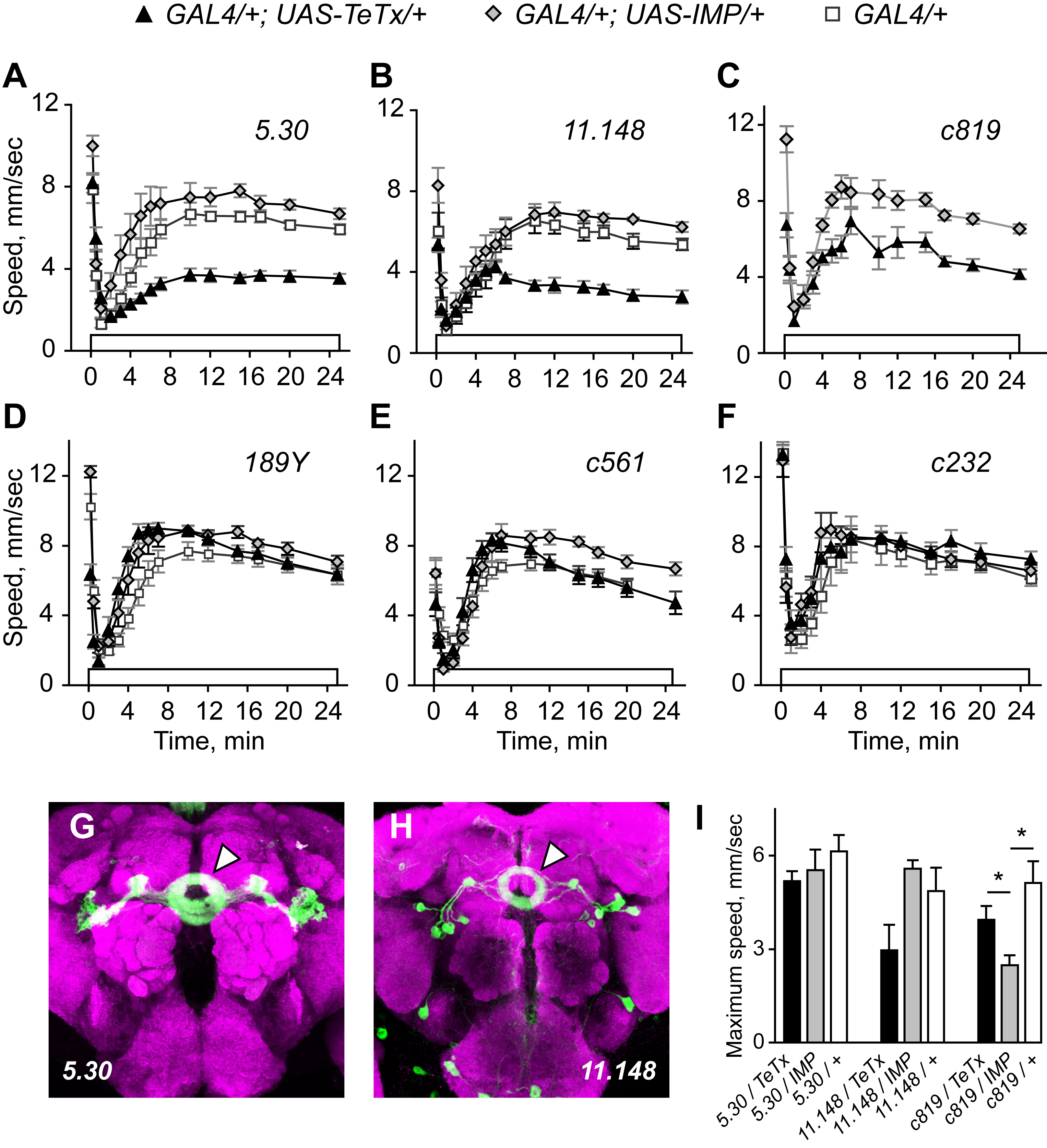

Supplement: Figure S4 — A–F. Locomotor activity profiles for the functional mapping of ellipsoid body neurons in ethanol-induced hyperactivity. Flies of the indicated genotypes were exposed to 47% ethanol vapor (open box on horizontal axis). GAL4 lines are indicated on each graph. G,H. Expression of the indicated GAL4 lines in R2/R4 neurons of the ellipsoid body (arrowhead), detected by GFP expression and counterstained with anti-Bruchpilot (nc82) to highlight the synaptic neuropil. I. Higher resolution analysis of startle magnitude for the GAL4 lines expressed in the R2/R4 neurons, corrected for pre-exposure locomotor activity levels. For 5.30 and 11.148, the olfactory startle responses were unaffected by TeTx expression (5.30: P = 0.4403, 11.148: P = 0.0556, 1 way ANOVA, n≥5). For c819, c819/+;UAS-IMP/+ was reduced (P = 0.0194, 1 way ANOVA, Tukey's multiple comparison test, n≥3). (5.76 MB TIF) [file pone.0009954.s005.tif]

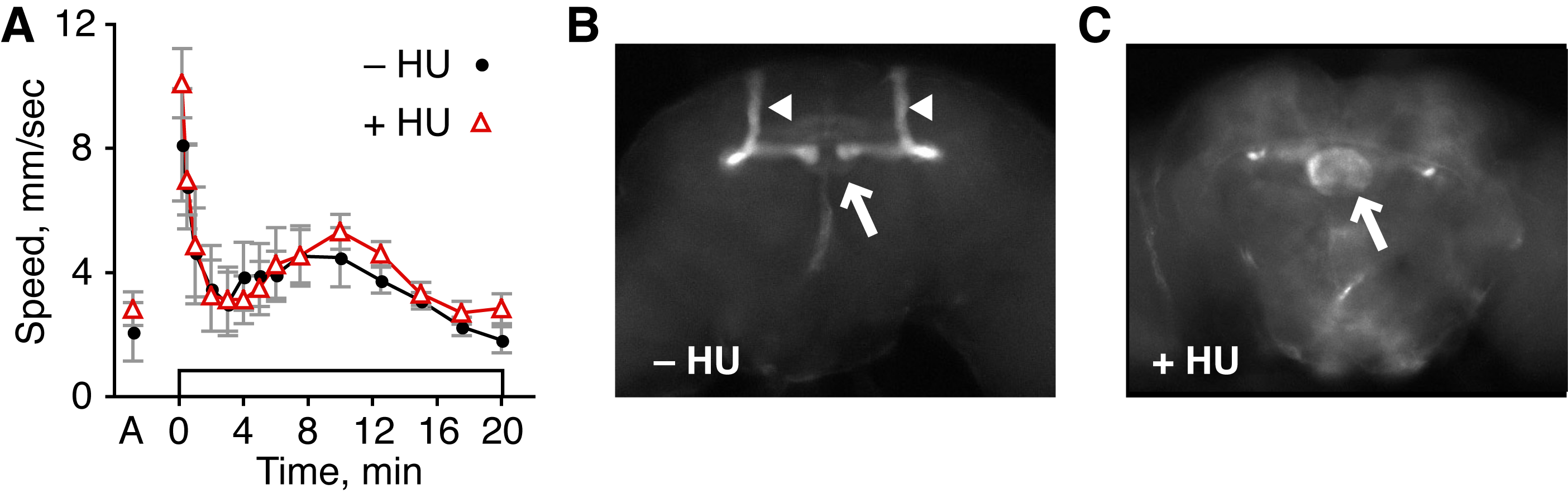

Supplement: Figure S5 — Mushroom body ablation. A. Ethanol-induced hyperactivity at 60% ethanol vapor with (+HU) and without (-HU) hydroxyurea treatment. Distance traveled 2–12.5 min did not differ (P = 0.0861, paired t-test, n = 4). B, C. Brains from control and HU treated flies, stained with anti-FasII to highlight the mushroom bodies (arrowheads) and the central complex ellipsoid body (arrow), which is unaffected by HU treatment. (1.33 MB TIF) [file pone.0009954.s006.tif]

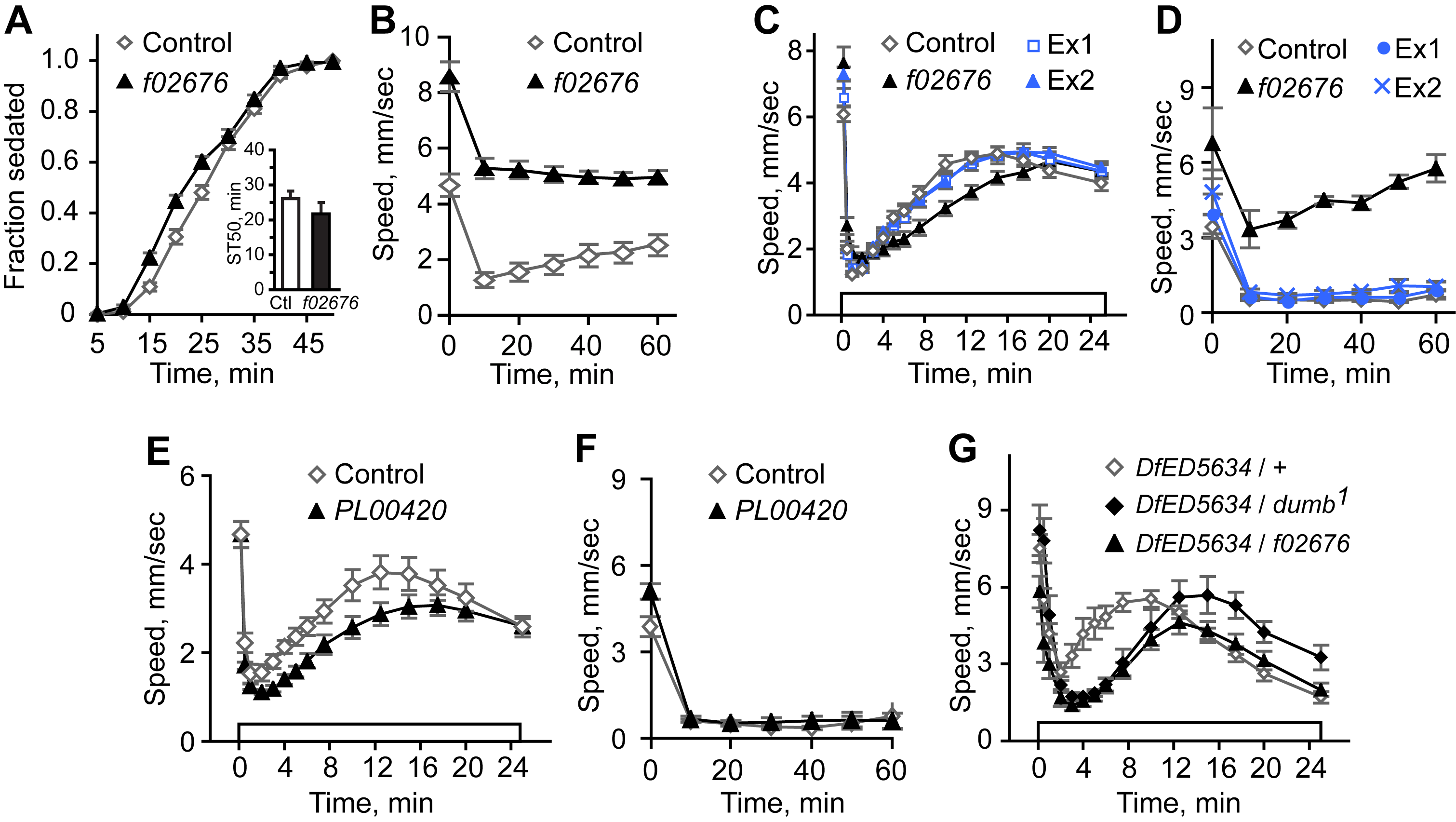

Supplement: Figure S6 — DopR mutant behavioral characterization. A. Ethanol-induced sedation was unaffected in f02676 homozygotes, measured as the loss of the ability to right. (60% ethanol vapor, P = 0.0945, 2 sample t-test, n = 10). B. Unstimulated locomotor activity, measured for 60 min immediately after flies were introduced into a 60×90×10mm Plexiglas box. DopRf02676 showed higher activity (distance traveled 10–60 min, P = 0.0009, 2 sample t-test, n = 10). C, D. Precise excision of f02676 reverts ethanol-induced hyperactivity and unstimulated activity behavioral phenotypes. C. Ethanol-induced hyperactivity for the genetic background control, f02676 homozygotes, and two independent precise excisions of f02676 (Ex1 and Ex2). D. Unstimulated activity for the same strains. Distance traveled from 10–60 min showed that f02676 is different from all other genotypes (P<0.001, 1 way ANOVA, Tukey's multiple comparison test, n = 5). E. Ethanol-induced hyperactivity in animals homozygous for PL00420. F. Unstimulated activity was unaffected in PL00420 homozygotes. G. Non-complementation for ethanol-induced hyperactivity by DfED5634, a deficiency that deletes the entire DopR locus, for f02676 and the dumb1 inversion allele. (0.65 MB TIF) [file pone.0009954.s007.tif]

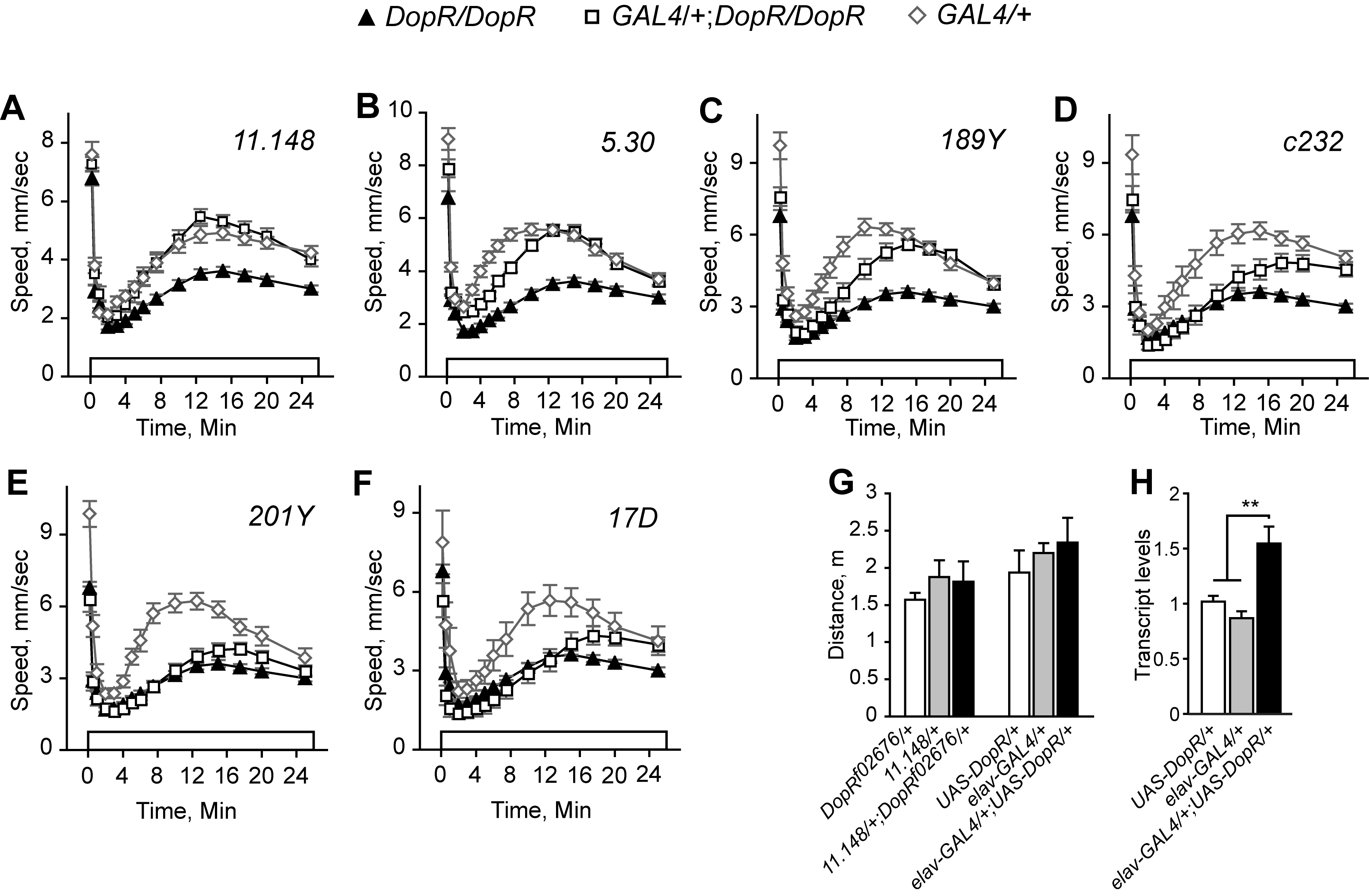

Supplement: Figure S7 — A–F. Locomotor activity profiles for the genetic rescue of the DopRf02676 mutant ethanol-induced hyperactivity phenotype. G. Expression of DopR utilizing the UAS sites in f02676 or a UAS-DopR transgene has no effect on ethanol-induced hyperactivity in flies that are either wild-type or heterozygous for DopR (left group: P = 0.7842, 1 way ANOVA, n = 8, right group: P = 0.575, 1 way ANOVA, n = 5). H. Increased DopR transcript levels when UAS-DopR is combined with the pan-neuronal elav-GAL4 (**P = 0.0062, 1 way ANOVA, Tukey's multiple comparison test, n = 3). (0.79 MB TIF) [file pone.0009954.s008.tif]
